# A connection between two ancient and essential cellular processes, iron-sulfur protein biogenesis and fatty acid synthesis, in *Escherichia coli*

**DOI:** 10.1128/mbio.00930-26

**Published:** 2026-06-15

**Authors:** Soufyan Fakroun, Guillaume Bouvier, Marouane Libiad, Emmanuel Séchet, Emmanuelle Bouveret, Frédéric Barras, Sarah Dubrac

**Affiliations:** 1 Department of Microbiology, Stress Adaptation and Metabolism in enterobacteria Unit, Institut Pasteur, Université Paris-Cité, CNRS UMR 555089, Paris, France; 2 Department of Structural Biology and Chemistry, Structural Bioinformatics Unit, Institut Pasteur, Université Paris-Cité, CNRS UMR 555089, Paris, France; University of California Irvine, Irvine, California, USA

**Keywords:** iron-sulfur cluster biogenesis, fatty acid biosynthesis, acyl carrier protein, *E. coli*

## Abstract

Iron-sulfur [Fe-S] clusters are ubiquitous cofactors of a wide array of structurally and functionally diverse proteins. Acyl carrier protein (ACP) is the universal factor required for fatty acid (FA) synthesis. In this study, in *E. coli*, we demonstrated that [Fe-S] and FA biosynthesis pathways are coordinated processes, driven by a physical interaction between ACP and the ISC [Fe-S] biogenesis machinery. Using bacterial two-hybrid assays, co-purification, and biochemical analyses, we demonstrated a molecular interaction between ACP and IscS, the ISC machinery cysteine desulfurase that provides sulfur for [Fe-S] cluster formation. Structural modeling and directed mutagenesis pinpointed the ACP-binding site in a region of IscS shared for interactions with other components of the ISC [Fe-S] biogenesis system, mainly Fdx and CyaY. At the cellular level, ACP depletion was found to disrupt ISC-dependent [Fe-S] cluster biogenesis, diminishing the activity of key [Fe-S]-dependent regulators (IscR, FNR, and NsrR) and enzymes (aconitase and biotin synthase). Moreover, FASII inhibition by triclosan, which perturbs ACP species homeostasis, also compromised the assembly of the [Fe-S]-dependent FNR regulator. Our findings underscore a functional link between [Fe-S] cluster biogenesis and fatty acid metabolism, with far-reaching unexplored intricacies of metabolic coordination and cellular homeostasis. Comparison with eukaryotic systems highlights a strong evolutionary driving force toward a link between [Fe-S] cluster and fatty acid biosynthesis in all living systems.

**IMPORTANCE:**

Cellular functions rely on interconnected metabolic pathways; however, many regulatory links remain unexplored. Iron-sulfur [Fe-S] clusters are cofactors of proteins driving fundamental cellular processes, from respiration to gene regulation. Our study uncovers a direct connection between [Fe-S] cluster biogenesis and fatty acid biosynthesis. We demonstrate the molecular connection between these two essential cellular processes to lie within the interaction between the acyl carrier protein (ACP), a shuttle of fatty acid biosynthetic intermediates, and IscS, the source of sulfur for [Fe-S] cluster assembly. Intriguingly, similar interactions between ACP and [Fe-S] building cysteine desulfurase have been observed in yeast and human models; however, these mechanisms rest on different molecular determinants. This points out the existence of a strong evolutive driving force toward establishing a link between [Fe-S] cluster and fatty acid biosynthesis in all living systems, with far-reaching implications for metabolic coordination and cellular homeostasis.

## INTRODUCTION

[Fe-S] clusters are metallic protein cofactors essential in nearly all living organisms. [Fe-S] proteins are involved in a wide range of cellular processes, such as respiration, metabolism, gene expression, RNA modification, lipid degradation, cell envelope biogenesis, vitamins, and cofactor biosynthesis (1). They facilitate a wide array of biochemical processes, from electron transfer to redox and non-redox chemical catalysis. *Escherichia coli* is predicted to synthesize more than 180 [Fe-S] proteins, with great diversity both in terms of structure and function. Hence, biogenesis of [Fe-S] cluster-containing proteins is essential for cell survival. This process is carried out by multi-protein machineries and occurs in two steps: the assembly of the [Fe-S] cluster and its delivery to apoprotein targets (2, 3). Five [Fe-S] biogenesis machineries were described: ISC in bacteria, archaea, and mitochondria; SUF in bacteria, archaea, chloroplasts, and organelles; MIS and SMS in both bacteria and archaea; and NIF in bacteria (4–6). The ISC machineries found in bacteria and mitochondria share significant similarities in functioning and composition. They include a cysteine desulfurase (IscS in bacteria and NFS1 in eukaryotes), a scaffold (IscU in bacteria and Isu in eukaryotes), a ferredoxin (Fdx in bacteria and Yah1 in eukaryotes), a frataxin (CyaY in bacteria and Ftn in eukaryotes), and a co-/chaperone duo (HscBA in bacteria and Hsp70/40 in eukaryotes). Briefly, the scaffold captures sulfur from cysteine desulfurase, and iron from an unidentified source assembles the [Fe-S] cluster, which is subsequently transferred to apo targets *via* [Fe-S] cluster carriers (7, 8).

In mitochondria, ACP interacts with the cysteine desulfurase NFS1 *via* ISD11, a member of the LYRM protein family characterized by a leucine-tyrosine-arginine (LYR) motif (9–11). Experimental evidence suggested that ACP enhances the ISC-dependent synthesis of [Fe-S] clusters (12). Interestingly, bacterial ACP was also reported to interact with the IscS cysteine desulfurase, despite the absence of ISD11 in bacteria. Indeed, ACP was found to co-purify with IscS (13), and later on, IscS was identified as a potential partner of ACP in a TAP-tag screen (14).

ACP provides acyl chains for the synthesis of phospholipids, lipopolysaccharides, biotin, and lipoate. The latter is an essential cofactor for enzymes of the TCA cycle, such as the pyruvate dehydrogenase (PDH), that is crucial for the energy homeostasis in both bacteria and eukaryotes (15, 16). To be active, ACP undergoes a post-translational maturation during which a phosphopantetheine group (4′-PP), derived from coenzyme A, is added to the side chain of a serine residue in position 36 of ACP. This modified form is known as holo-ACP, which represents the active form of the carrier, capable of binding a malonyl chain (from malonyl-CoA) through *trans*-esterification to the thiol group at the 4′-PP terminus (Fig. S1). This acylated-ACP then interacts with the enzymes of the FASII pathway (Fab enzymes) that perform acyl chain elongation. Structurally, ACP is a small three-α-helix bundle protein. The serine 36 is located in the α_2_ helix, which mediates interactions with the Fab enzymes set (17). Beyond its interaction with the cysteine desulfurase IscS, ACP has also been found to interact with SpoT, a (P)ppGpp hydrolase, and MukB, a protein involved in chromosome partitioning during cell division (14, 18).

In the present study, we investigated the molecular basis and the physiological significance of the interaction between IscS and ACP. By using a biomolecular interaction modeling (Boltz) as well as a bacterial two-hybrid assay, we identified key residues involved in the IscS/ACP interaction. Moreover, we demonstrated that ACP acts as a positive effector of the activity of several diverse [Fe-S] protein targets in an ISC-specific manner. We propose that ACP availability (and/or its acylation state) helps to coordinate [Fe-S] cluster and fatty acid homeostasis, thereby adapting the efficiency of key cellular pathways to meet cellular needs.

## RESULTS

### A model-based prediction of the IscS-ACP interaction

**Fig 1:**
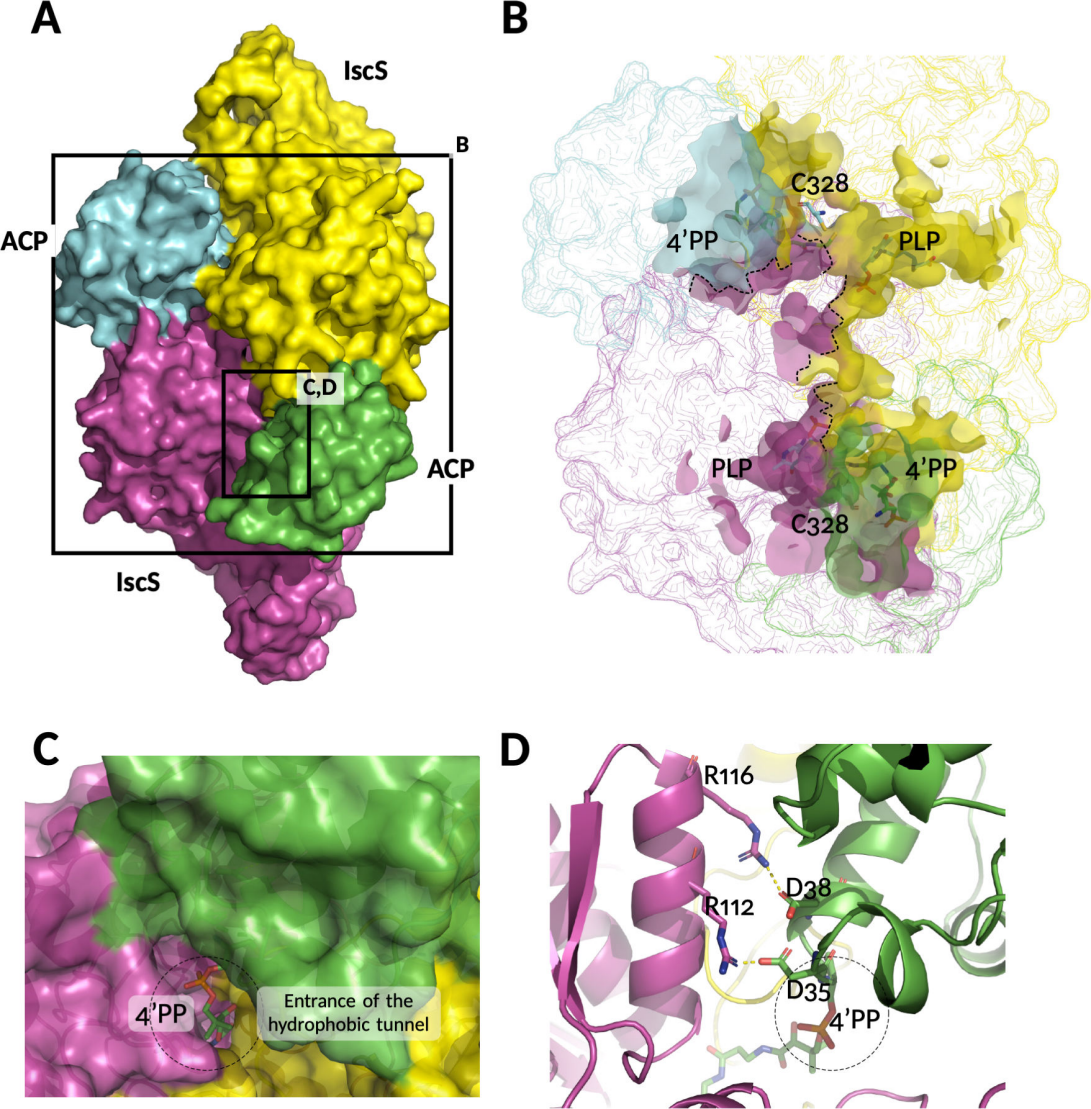
Modeling of the ACP-IscS complex. (**A**) Overall structure of the ACP_2_-IscS_2_ complex showing surface interaction between an IscS dimer (magenta and yellow) and two ACP monomers (blue and green). Frameworks delineate the region of the structure represented in B, C, and D as indicated. (**B**) Inner view of the hydrophobic tunnel formed at the interface (dotted line) of the two IscS monomers. The 4′-PP arm is penetrating the tunnel, where the PLP is also present. The catalytic cysteine residue of IscS is also indicated. The tunnel is depicted in surface representation, while the complex is shown as a contour, keeping the same orientation as in A. (**C**) Zoom in the ACP-IscS interaction zone shown as a surface with the 4′-PP arm fitting into the hydrophobic tunnel at the interface between the two IscS monomers. (**D**) Salt-bridge interactions detected at the interface between the ACP (green) and the IscS protein (magenta). Key amino acids involved in the interaction are shown as sticks, highlighting ACP residues Asp_35_ and Asp_38_, and IscS residues Arg_112_ and Arg_116_. The ACP-bound 4′-PP arm is also indicated, and the entrance of the hydrophobic tunnel is outlined with a dotted ring.

Occurrence of an interaction between ACP and IscS was suggested by protein-protein interaction methods (13, 14). Therefore, we wished to investigate the validity of this interaction by modeling the ACP-IscS complex using Boltz-2, a diffusion-based generative AI model that predicts 3D protein and biomolecular complex structures from amino acid sequences (19). The resulting model showed that a dimer of IscS interacts with two ACP monomers (Fig. 1A). The phosphopantetheine group of holo-ACP is predicted to fit into a hydrophobic tunnel formed at the interface between the two IscS monomers (Fig. 1B and C). It is noticeable that the PLP (pyridoxal 5′-phosphate) cofactor of IscS also locates in this tunnel, while the catalytic residue Cys_328_ is oriented toward the 4′-PP arm (Fig. 1B). Moreover, a positively charged region of IscS is predicted to interact with a negatively charged region of ACP, involving the pair IscS Arg_112_-ACP Asp_35_ and the pair IscS Arg_116_-ACP Asp_38_ (Fig. 1D). This analysis gave further support to the notion that *E. coli* IscS and ACP interact directly.

### Molecular characterization of the IscS-ACP interaction

**Fig 2:**
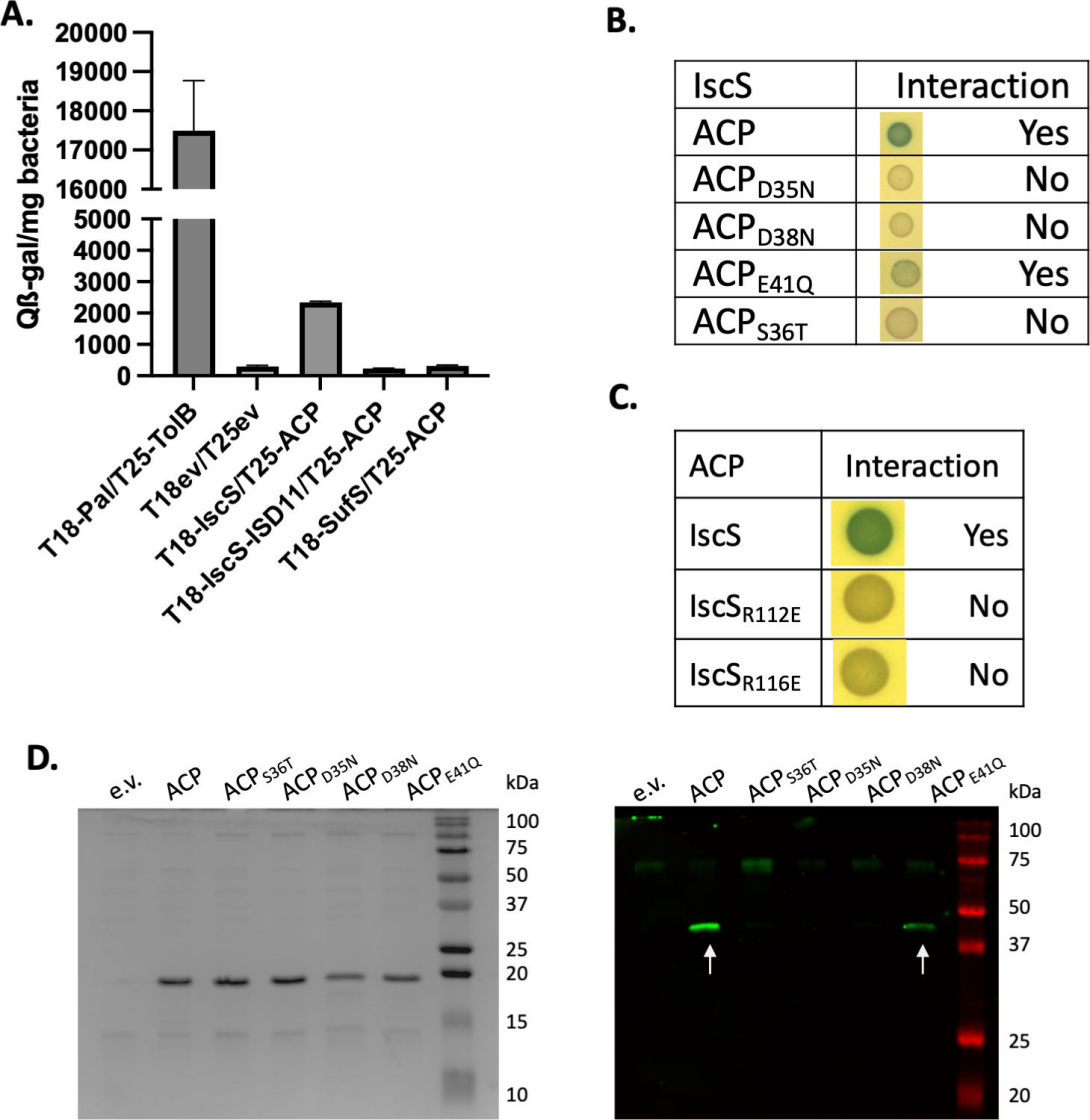
The IscS cysteine desulfurase forms a complex with ACP. (**A**) The ACP protein was tested for pairwise interactions with the IscS and SufS cysteine desulfurases using the BACTH assay. Translational fusions of tested proteins with T25 or T18 adenylate cyclase domains were co-transformed into BTH101 strain and ß-galactosidase activity assay for the indicated pairs was performed. For testing ISD11 interferences, the human *isd11* was co-expressed with the T18-IscS fusion. A positive control of interaction using the Tol and Pal proteins was included, as well as a negative control with a strain containing the pT18 and pT25 empty vectors. Data are the mean of data obtained from three biological replicates, and error bars are standard errors of the mean. ***, *P* < 0.001, one-way ANOVA with post hoc multiple comparisons performed via the Dunnett test. (**B**) Role of negative residues of ACP (Asp_35_, Asp_38_, and Asp_41_), as well as the phosphopantetheine binding site of ACP (Ser_36_) in ACP-IscS interactions. Fusions of ACP variant alleles (D_35_N, D_38_N, E_41_Q, S_36_T) with T25 were obtained by PCR site-directed mutagenesis of the parent pT25-ACP plasmid. BTH101 cells co-transformed with pT18-IscS and pT25-ACP and ACP variants were dropped (3 µL) on LB plates containing X-gal and IPTG and incubated for 24 h at 30°C. (**C**) Role of positive residues of IscS (Arg_112_ and Arg_116_) in ACP-IscS interactions. Fusions of IscS variant alleles (R_112_E, R_116_E) with T18 were obtained by site-directed mutagenesis of the parent pT18-IscS plasmid. (**D**) Each pairwise interaction was confirmed using a co-purification assay. CBP-ACP (or ACP variants as indicated) were produced from plasmids derived from the pBAD-CBP vector. Plasmids were transformed into the MG1655 strain. Resulting strains were grown in LB until OD_600nm_ 0.5 and exposed to 0.01% arabinose for 60 min at 37°C. After protein extraction and CBP protein fusions purification (see Materials and Methods), samples were analyzed on a 12% SDS-PAGE (left panel) showing CBP fusion proteins and by western blot with anti-IscS antibodies (right panel). White arrows indicate IscS detection.

In order to assess the model-based prediction above, the interaction between ACP and IscS was investigated using a series of different molecular approaches. First, we used the Bacterial Adenylate Cyclase Two-Hybrid (BACTH) system (20). ACP was fused with the T25 subunit of *Bordetella pertussis* adenylate cyclase, while IscS was fused with the T18 subunit. Co-transformation of *E. coli* BTH101 cells with these constructs (T25-ACP and T18-IscS) resulted in high level of β-galactosidase activity (Fig. 2A), as predicted if the two proteins interact. Two types of specificity controls were performed. First, we took advantage of the capacity of human ISD11 to bind bacterial ACP (21) and reasoned that overexpression of ISD11 should compete with IscS for binding to ACP. We co-expressed human ISD11 and indeed observed a loss of interaction between ACP and IscS (Fig. 2A). Second, we tested whether SufS, another *E. coli* cysteine desulfurase, was able to bind ACP. No interaction was observed between T25-ACP and T18-SufS, reinforcing the specificity of the ACP-IscS interaction (Fig. 2A).

Next, using site-directed mutagenesis, we generated variants of ACP that were modified on the residues predicted to be involved in the ACP-IscS interaction. The T25-ACP_D35N_ and T25-ACP_D38N_ were generated and tested for interaction with IscS using the BACTH assay. We also included the T25-ACP_E41Q_ variant as a control to assess the effect of modifying another negative residue in the same region. Both T25-ACP_D35N_ and T25-ACP_D38N_ failed to bind to IscS, while a residual level of β-galactosidase activity was observed for the T25-ACP_E41Q_/T18-IscS pair (Fig. 2B). Additionally, the T25-ACP_S36T_ variant, in which the critical serine (S) residue at position 36 was changed to threonine (T), showed no interaction with IscS (Fig. 2B). This observation suggested that the serine in position 36 of ACP contributes to the interaction with IscS, presumably by allowing the 4′-PP arm to interact with IscS, as predicted by the model above. Conversely, we tested the interaction of T25-ACP with IscS variants. Our data indicated that T25-ACP did not interact with T18-IscS_R112E_ or T18-IscS_R116E_ (Fig. 2C).

Last, we used a co-purification-based approach. A Calmodulin-binding protein (CBP)-tagged ACP recombinant protein was produced from a plasmid. Both wild-type CBP-ACP and CBP-ACP variants were purified from cells (Fig. 2D, left panel). Endogenous IscS was co-purified with wild-type CBP-ACP and with the CBP-ACP_E41Q_ variant, but with none of the CBP fusions with ACP_D35N_, ACP_D38N_, or ACP_S36T_ variants (Fig. 2D, right panel). Consistent with the BACTH data, these results strongly supported the notion that ACP D_35_, D_38_, and S_36_ residues are critical for the interaction with IscS.

**Fig 3:**
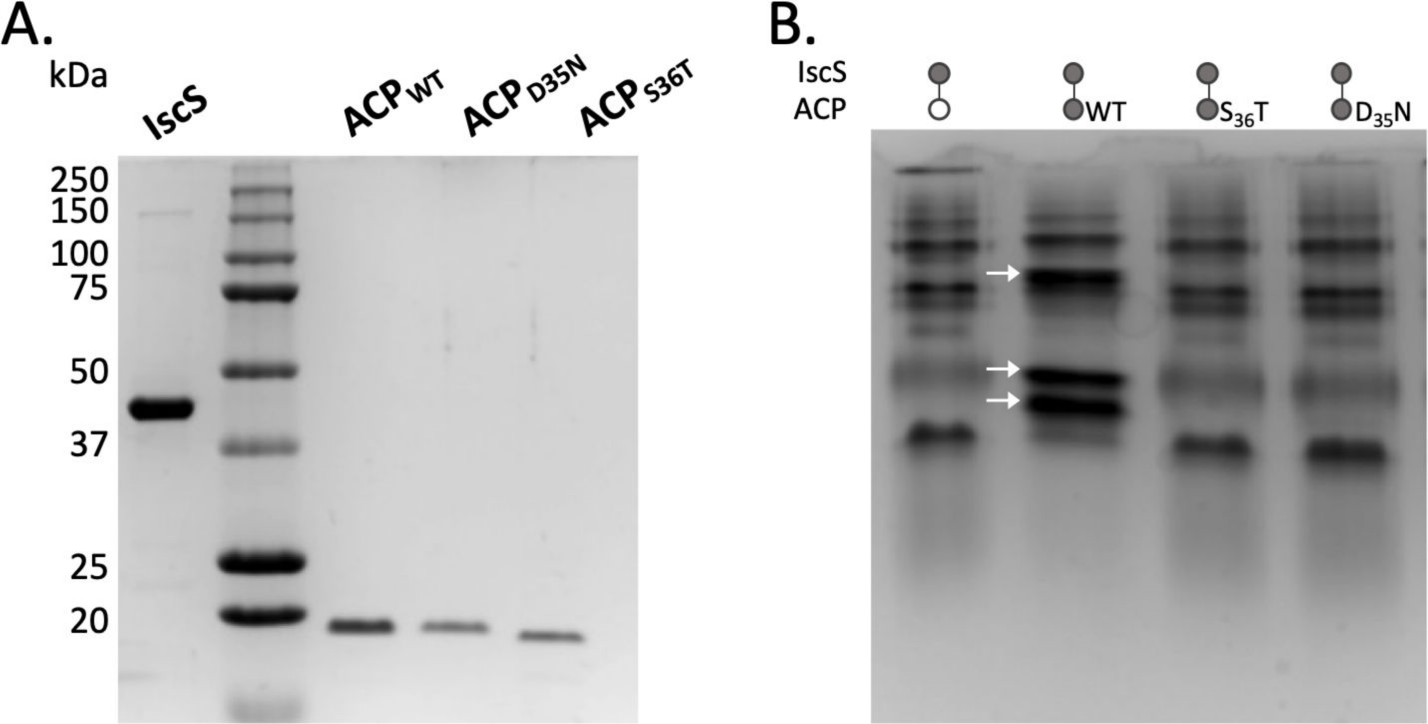
Direct *in vitro* interaction between ACP and IscS. **A**) SDS-PAGE of the purified 6His-IscS, 6His-ACP (ACP_WT_), and the ACP variants 6His-ACP_D35N_ and 6His-ACP_S36T_. (**B**) Native PAGE showing 6His-IscS alone (15 µM) or mixed with 6His-ACP (30 µM for either wild type (WT), D_35_N, or S_36_T variants) as indicated. Prior to electrophoresis, protein mixtures were incubated in purification buffer for 20 min at 37°C (see Materials and Methods section). Loaded protein solutions are indicated above the gel picture. White arrows indicate shifted IscS bands upon incubation with ACP_WT_.

In parallel, we purified 6His-tagged versions of ACP (wild-type, D_35_N, and S_36_T) along with 6His-IscS using nickel affinity chromatography (Fig. 3A). A mixture of IscS with these different ACPs was analyzed by native PAGE and Coomassie Blue staining. The migration pattern of IscS alone revealed several bands, likely corresponding to different oligomeric forms of IscS (Fig. 3B, first lane). Incubation of IscS with wild-type ACP resulted in a shift in the migration pattern, indicating an interaction between IscS and ACP (Fig. 3B, white arrows). In contrast, no such shift was observed when IscS was incubated with the ACP S_36_T or D_35_N variants, further confirming the loss of interaction between these mutants and IscS (Fig. 3B).

Together, these experiments demonstrated a direct and specific interaction between ACP and IscS that critically depends on ACP Ser_36_, Asp_35_, and Asp_38_, with the latter two residues likely forming salt bridges with Arg_112_ and Arg_116_ of IscS.

### Acylated ACP enhances the cysteine desulfurase activity of IscS

**Fig 4:**
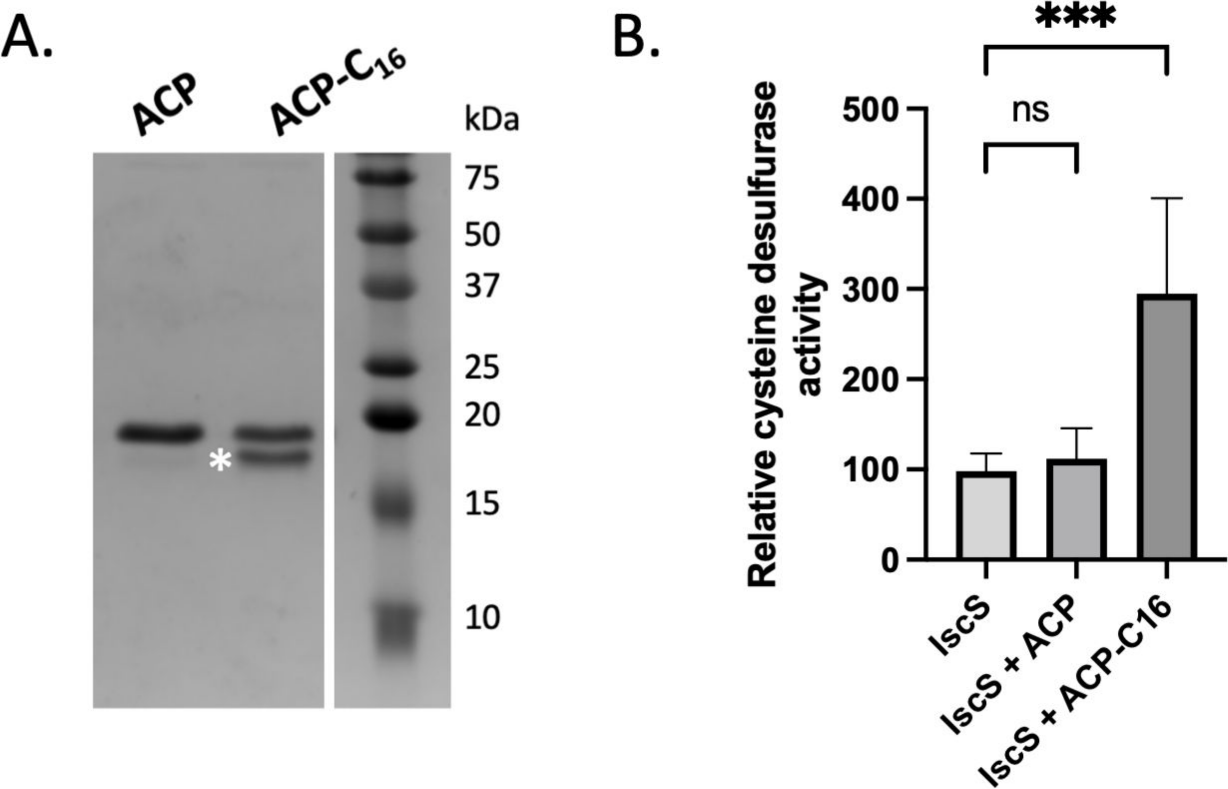
Acylated ACP enhances the cysteine desulfurase activity of IscS. (**A**) Purified 6His-ACP was acylated using palmitate (C16:0) and pure Aas acyl-ACP synthase to catalyze the acylation. SDS-PAGE shows the purified 6His-ACP and acylated 6His-ACP, with the acylated form indicated by an asterisk. (**B**) Cysteine desulfurase (CD) activity was measured using the methylene blue assay. CD activity was assessed for IscS alone and for IscS pre-incubated with either purified 6His-ACP or acylated 6His-ACP as described in A). The results are presented as relative CD activity normalized to the activity of IscS alone. Values and error bars represent the means and standard errors of the mean of six independent measurements. Statistical significance was determined using one-way ANOVA with post hoc multiple comparisons performed via the Dunnett test; ns: not significant; ***, *P* < 0.001.

We next investigated whether ACP had an impact on the cysteine desulfurase activity of IscS. The purified ACP preparation was expected to comprise a mixture of apo-ACP, holo-ACP (phosphopantetheine-bound ACP), and a minor fraction of acylated ACP (22). Therefore, by using purified acyl-ACP synthetase (Aas), we acylated ACP with palmitic acid (C16:0). Upon SDS-PAGE analysis, the acylated ACP (ACP-C16) displayed a faster migration compared to the unmodified ACP, indicating successful acylation (Fig. 4A) (23). Only a portion of the purified ACP could be acylated, likely due to the proportion of holo-ACP available in the ACP purification. In parallel, we assessed whether ACP variants previously shown to lose the capacity to bind IscS (Fig. 3) were susceptible to acylation. In contrast to wild-type ACP, ACP_S36T_ and ACP_D35N_ maintained identical migration patterns in the presence or absence of palmitic acid, indicating that neither mutant could be acylated (Fig. S2). We then assayed IscS desulfurase activity, which increased 3-fold upon incubation with ACP-C16 but remained unchanged with non-acylated ACP (Fig. 4B).

These results showed that acylated-ACP enhances IscS cysteine desulfurase activity.

### Decrease in ACP levels leads to [Fe-S]-dependent phenotypes

The *acpP* gene, which encodes ACP, is essential, thereby precluding the use of a null mutant to investigate the effect of ACP levels on [Fe-S] cluster biogenesis *in vivo*. Therefore, we used CRISPR-based RNA interference (CRISPRi) to downregulate ACP levels. The CRISPRi system used in this study consists of two plasmids: (i) the psgRNA plasmid, which produces a guide RNA under the control of a constitutive promoter, and (ii) the pdCas9 plasmid, wherein the expression of dCas9 is controlled by the TetR-regulated promoter, Ptet, and can be induced by anhydrotetracycline (AnTet) (24). We designed a guide RNA (gRNA-*acp*) complementary to a portion of the *5'UTR* of *acpP* to generate the psgRNA-*acp* plasmid (Fig. S3A). To clarify, “e.v.” refers to strains containing the pdCas9 and psgRNA plasmids, while “CRISPRi-acp” indicates strains that carry the pdCas9 and psgRNA-*acp* plasmids. Induction of dCas9 expression with 500 ng/mL of AnTet prevented bacterial growth of the CRISPRi-acp strain (Fig. S3B). Therefore, we reduced the concentration of the dCas9 inducing AnTet down to 0.1 ng/mL. This yielded a 5-fold decrease in *acpP* expression in the CRISPRi-acp strain compared to the e.v. control (Fig. S3D). We checked that under these conditions, there was no effect on bacterial growth and that the fitness of the CRISPRi-acp was the same as the e.v. strain (Fig. S3C). We also examined whether reduced *acpP* expression induced a general stress response. We monitored two reporters of the general stress response: *rpoS* itself and *dps*, a RpoS-induced gene. Detection levels of both RpoS and Dps-SPA were comparable between the CRISPRi-acp strain and the empty vector (e.v.) control (Fig. S4), showing that in the conditions used, CRISPRi-mediated downregulation of *acpP* did not induce a general stress response.

**Fig 5:**
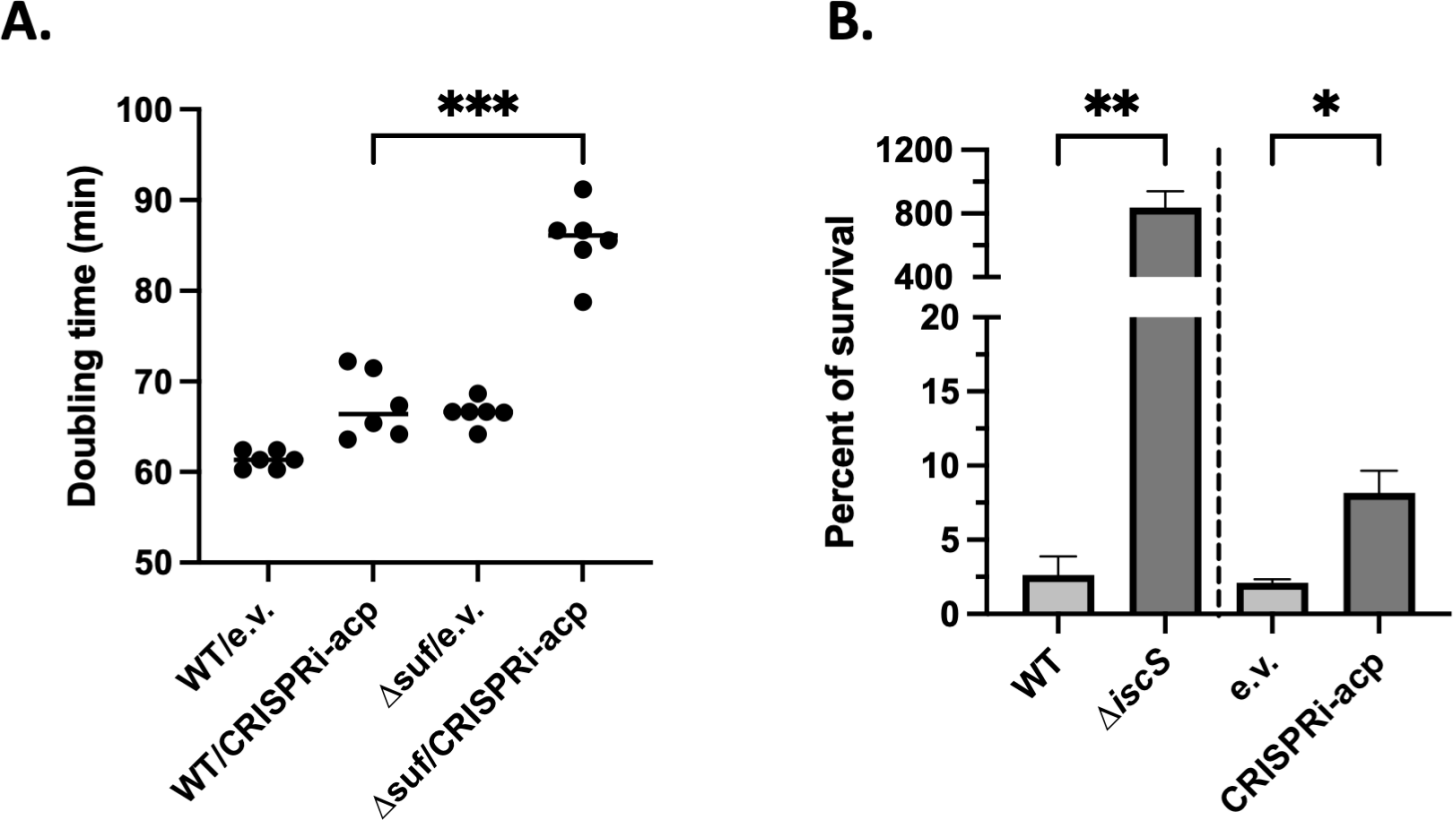
Reduced ACP levels affect the efficiency of the ISC machinery. (**A**) Growth of WT/e.v., WT/CRISPRi-acp, a ∆*sufBCD*/e.v., and ∆*sufBCD*/CRISPRi-acp strains was done in LB medium with 0.1 ng/mL AnTet, and OD_600nm_ was measured using a Tecan microplate reader. WT was the MG1655 strain. Doubling times were calculated during the exponential growth phase. The results of six biological replicates are presented with average growth rates and their respective variations. ***, *P* < 0.001, unpaired Student’s *t*-test. (**B**) Strains were grown in LB for MG1655 (WT) and ∆*iscS* and LB- AnTet 0.1 ng/mL for the e.v. and CRISPRi-acp strains until OD_600nm_ around 1. Survival of strains was measured after 3 h of treatment with gentamicin (5 µg/mL) by counting colony-forming units (CFU) and normalized to the time at which the antibiotic was added to calculate the rate of survival. The data presented are the means of four biological replicates. **, *P* < 0.01; *, *P*< 0.05, unpaired Student’s *t*-test.

Next, we investigated the effect of reduced ACP levels on bacterial fitness in a *ΔsufBCD* strain, wherein [Fe-S] cluster formation is entirely dependent on the ISC pathway. We observed that the growth rate of the *ΔsufBCD/*e.v. strain was 66.55 ± 1.58 min, while the *ΔsufBCD/*CRISPRi-acp strain exhibited a doubling time of 85 ± 4.51 min. Conversely, lowering ACP levels had no noticeable effect in a wild-type background (MG1655, WT) as doubling times were 61 ± 1 and 67 ± 4 min for WT/e.v. and WT/CRISPRi-acp, respectively (Fig. 5A). Next, we assessed the sensitivity of WT/e.v. and WT/CRISPRi-acp to aminoglycoside antibiotics, a phenotype that is controlled by the ISC system efficiency (25). The e.v. strain exhibited high sensitivity to gentamicin, an aminoglycoside, with only 2% survival after 3 h of treatment with 5 µg/mL of gentamicin. In contrast, the CRISPRi-acp strain showed enhanced tolerance, with over 8% survival, suggesting that reduced ACP levels impaired ISC activity, leading to enhanced aminoglycoside tolerance (Fig. 5B, right panel). As a control, we also tested a Δ*iscS* strain, which showed a high level of tolerance (Fig. 5B, left panel).

Overall, these results indicated that a reduction in ACP levels negatively affected ISC efficiency.

### Transcriptional activity of [Fe-S]-dependent regulators is stimulated by ACP

To further assess the impact of reduced ACP levels on [Fe-S] cluster biogenesis *in vivo*, we tested whether the activity of several well-characterized [Fe-S] cluster regulators was affected by reducing the ACP levels. Specifically, the activity of IscR, FNR, and NsrR, which are [Fe-S] bound transcriptional regulators involved in [Fe-S] homeostasis, anaerobic adaptation, and nitrosative stress responses, respectively, was assayed (26).

**Fig 6:**
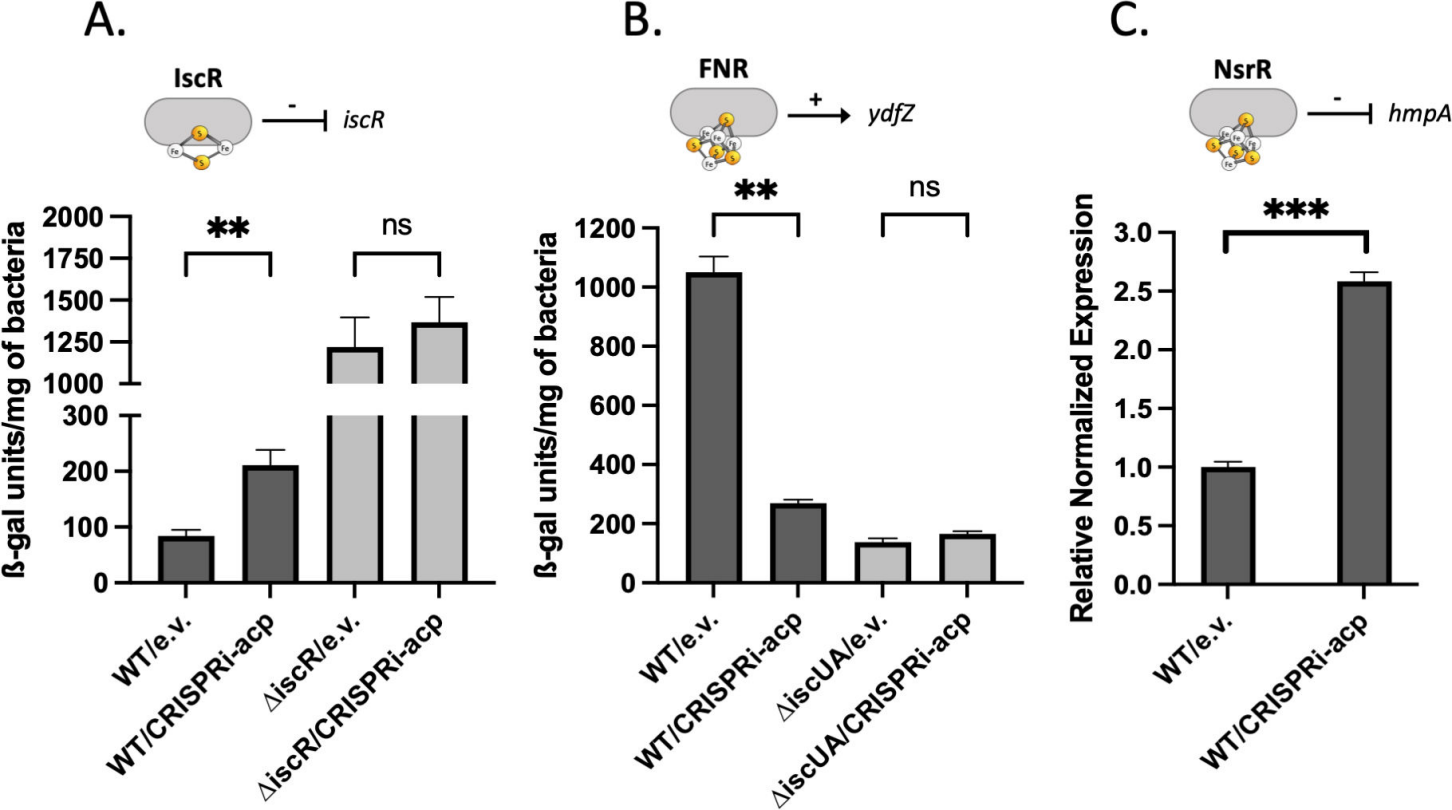
ACP levels interfere with the activity of [Fe-S] regulators. (**A**) Activity of the *iscR* promoter was assessed by measuring the β-gal activity (Miller units) of the P*iscR-lacZ* strains carrying pdCas9 and psgRNA (e.v.) or pdCas9 and psgRNA-*acp* (CRISPRi-acp) in either a WT (FBE005) background or a ∆*iscR* (FBE008) background. Strains were grown in LB with 0.1 ng/mL of AnTet and 62 µM of dipyridyl until OD_600nm_ = 2 before β-gal activity was measured. (**B**) Activity of the *ydfZ* promoter was assessed by measuring the β-gal activity (Miller units) of the P*ydfZ-lacZ* strains carrying the indicated plasmids in either a WT background (FBE204), or a ∆*iscUA* background (FBE596). Strains were grown in LB with 0.1 ng/mL of AnTet until OD_600nm_ = 2 before β-gal activity was measured. (**C**) Expression of *hmpA* in a WT (MG1655) background carrying pdCas9 and psgRNA (e.v.) or pdCas9 and psgRNA-*acp* plasmids (CRISPRi-acp). Strains were grown in LB with 0.1 ng/mL of AnTet until OD_600nm_ = 1 before RNA extraction. Gene expression was measured using quantitative real-time PCR (qRT-PCR). Expression levels were normalized using 16S rRNA as an internal standard and presented as the n-fold change of the CRISPRi-acp strain compared to the e.v. strain. Results are presented as the mean of 4 replicates, and standard errors of the mean are indicated. Statistical significance was determined using one-way ANOVA with post hoc multiple comparisons performed via the Dunnett test; ***, *P* < 0.005; **, *P* < 0.01; ns, no significant difference.

IscR is active under two forms: the holo-IscR (containing a [2Fe-2S] cluster), which represses its own expression among other targets, and the apo-IscR, which regulates another set of genes (27). We utilized a P*iscR-lacZ* chromosomal fusion to monitor the efficiency of IscR maturation. In strains grown in rich LB medium, the P*iscR-lacZ* fusion exhibited less than 20 units/mg of β-galactosidase, supporting the notion that IscR was predominantly in its repressing holo-form (Fig. S5). Upon addition of 2,2′-dipyridyl (DIP), an iron chelator, expression increased in a dose-dependent manner, reflecting a shift from the holo- to the apo-form of IscR and the associated alleviation of repression (Fig. S5). The prediction was that lowering the level of ACP should lower the efficiency of ISC-mediated maturation of IscR, which will eventually lead to the de-repression of the P*iscR-lacZ* fusion. The experiment was run in the presence of 62.5 µM DIP, a condition in which Fe-limitation is modest and likely allows the presence of both apo- and holo-forms of IscR. In this condition, depletion of ACP *via* CRISPRi (CRISPRi-acp) resulted in more than a 2-fold increase in P*iscR-lacZ* expression (Fig. 6A, dark gray plots). Importantly, in an ∆*iscR* strain, P*iscR-lacZ* expression was significantly higher, reflecting the absence of the IscR repressor. It was also insensitive to changes in ACP levels, demonstrating that the ACP-mediated effect on *iscR* expression was IscR-dependent (Fig. 6A, light gray plots).

FNR is active upon binding a [4Fe-4S] cluster and is inactivated by oxygen (28). We measured P*ydfZ-lacZ* activity as a proxy for FNR activity. The *ydfZ* promoter remains activated by FNR under aerobic conditions, albeit at a reduced level (29, 30). While the WT strain exhibited high levels of β-galactosidase activity, depletion of ACP led to a significant decrease in activity, reducing it by approximately 5-fold (Fig. 6B, dark gray plots). In contrast, in a *ΔiscUA* background, where FNR maturation is impaired, P*ydfZ-lacZ* activity was reduced compared to the WT, and varying ACP levels had no further effect on this strain (Fig. 6B, light gray plots). This result supported the notion that ACP depletion impacted ISC-dependent maturation of FNR.

NsrR is a [4Fe-4S] containing regulator that plays a crucial role in the bacterial response to nitrosative stress. Under its holo-form, NsrR acts as a repressor of NO defense-related genes, such as *hmpA* (31). To investigate the impact of ACP on NsrR activity, we monitored *hmpA* expression by qRT-PCR. Upon reduced ACP amount (CRISPRi-acp), we observed a more than 2-fold increase in *hmpA* expression (Fig. 6C), consistent with the notion that ACP enhanced NsrR repression activity.

SoxR binds a [2Fe-2S] cluster. It is activated under exposure to redox cycling drugs such as paraquat through redox state change of its [Fe-S] cluster (from +1 to +2). Importantly, under paraquat exposure, SoxR acquires its [Fe-S] cluster exclusively through the SUF system (32, 33). Using a P*soxS-lacZ* transcriptional fusion as a reporter for SoxR activity in the presence of paraquat, we observed that the induction of *soxS* expression was not altered in a strain with reduced ACP levels (Fig. S6A). This showed that ACP depletion did not affect [Fe-S] biogenesis carried out by the SUF system.

Together, these results showed that ACP enhanced maturation of [Fe-S] cluster-containing regulators, such as IscR, FNR, and NsrR, and strengthened the conclusion that ACP had a positive effect on ISC-dependent production of [Fe-S] clusters. Importantly, it had no effect on SUF-dependent [Fe-S] cluster production.

### Activity of [Fe-S]-dependent enzymes is stimulated by ACP

To investigate whether ACP-positive effect on ISC activity extends to [Fe-S]-dependent enzymes, we analyzed the activity of aconitase and biotin synthase.

**Fig 7:**
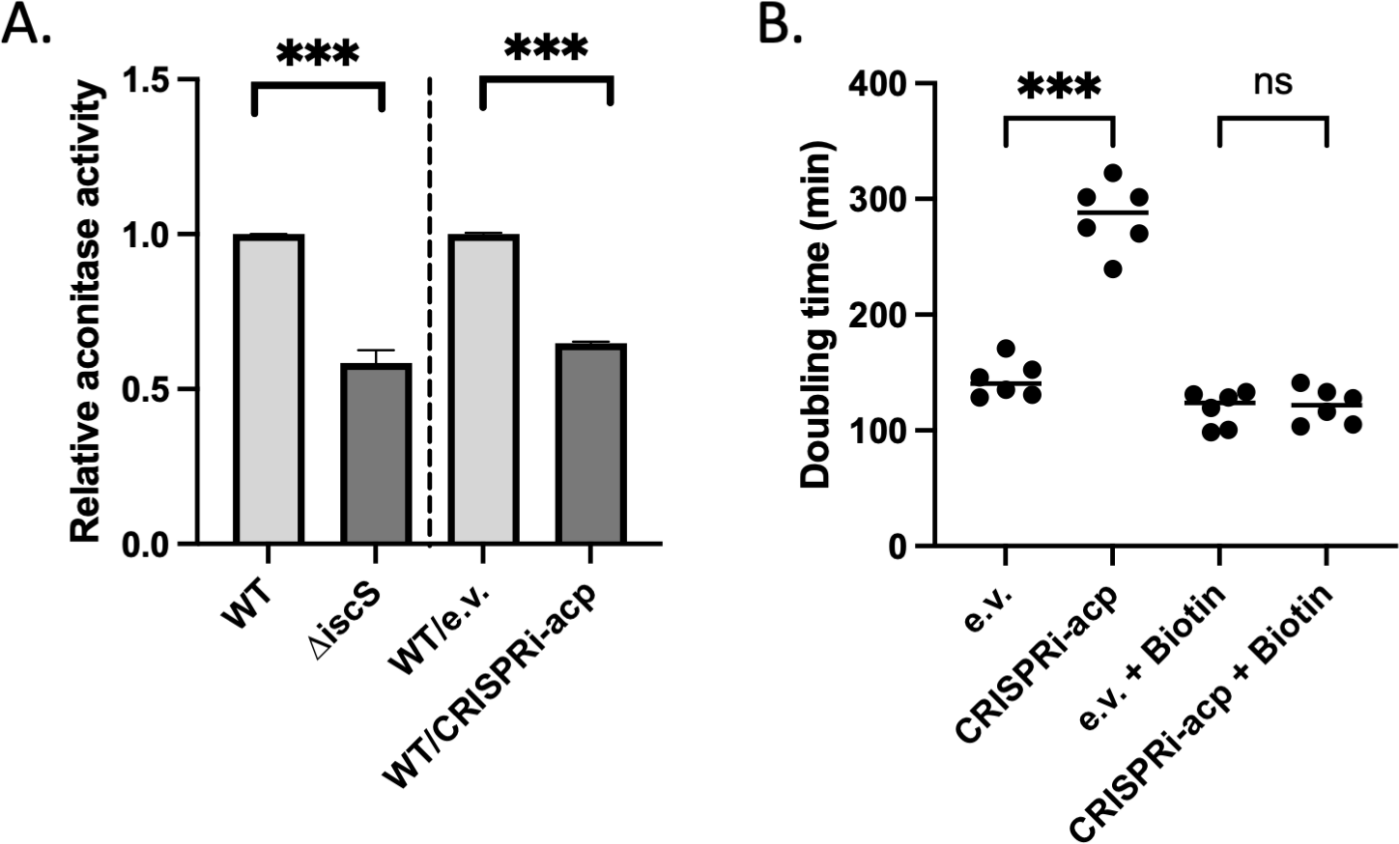
ACP affects the activity of aconitase and biotin synthase [Fe-S] enzymes. (**A**) Wild-type strain (MG1655) and ∆*iscS* derivative were grown in LB, and wild-type carrying pdCas9 and psgRNA (e.v.) or psgRNA-*acp* (CRISPRi-acp) were grown in LB with 0.1 ng/mL of AnTet, until OD_600nm_= 2. Aconitase activity was measured on crude extracts immediately after sonication (see Materials and Methods). Results are presented as relative aconitase activity compared to either the wild-type (left panel) or the wild-type carrying pdCas9 and psgRNA (right panel). Values represent the mean of at least four biological replicates, and standard errors of the mean are indicated. Statistical significance was determined using one-way ANOVA with post hoc multiple comparisons performed via the Dunnett test; ***, *P*<0.005. (**B**) Bacterial growth rate was used as a readout of biotin synthase activity. Mutant strain with a deletion in *bioD* carrying pdCas9 and psgRNA (e.v.) or psgRNA-*acp* (CRISPRi-acp) were grown in M9 minimal medium supplemented with dethiobiotin (0.1 mM) as a substrate of BioB. Biotin (0.1 mM) was added in half of the cultures where indicated. Growth rates are presented as the mean doubling time of six biological replicates for each strain and condition. Statistical significance was determined using one-way ANOVA with post hoc multiple comparisons performed via the Dunnett test; ***, *P* < 0.005; ns, no significant difference.

Aconitase activity, which results from the activity of two [4Fe-4S] enzymes AcnA and AcnB, exhibited a 40% reduction in activity in an ∆*iscS* background, a result consistent with prior studies (Fig. 7A, left panel) (34). CRISPRi-mediated down-expression of *acpP* reduced aconitase activity by 30% compared to the empty vector control (Fig. 7A, right panel), highlighting a positive role of ACP in supporting aconitase activity.

BioB is an Fe-S enzyme essential for the final step of the synthesis of biotin, an essential vitamin (Fig. S7A and B). In a Δ*bioD* strain, biotin synthesis depends on BioB-catalyzed conversion of dethiobiotin (DTB) to biotin, fitness in DTB-supplemented minimal medium served as an indirect measurement of BioB activity (35). Downregulation of *acpP* (CRISPRi-acp) in the Δ*bioD* background resulted in a doubling time of 288 min, a 2-fold increase compared to the empty vector strain (140 min) in minimal medium with DTB. This fitness defect was fully rescued by supplementing biotin, confirming that reduced growth was due to impaired BioB activity (Fig. 7B).

Importantly, ACP knockdown did not affect the activity of a non-[Fe-S] enzyme, i.e., endogenous β-galactosidase (Fig. S6B).

Together, these results demonstrated that ACP is a positive effector of [Fe-S]-dependent enzymes aconitase and biotin synthase, likely by enhancing their maturation.

### tRNA thiolation is not altered by varying ACP levels

IscS also acts as a sulfur donor for tRNA modification (36). Therefore, we investigated whether ACP also influences IscS-dependent tRNA thiolation catalyzed by MnmA, TtcA, MiaB, and ThiI. Our results indicated no significant changes in tRNA thiolation levels under ACP depletion (Fig. S8).

This suggested that downregulation of ACP has no effect on the sulfur transferase activity of IscS required for tRNA thiolation.

### FASII-targeted antibiotic treatment decreases Fe-S biosynthesis

**Fig 8:**
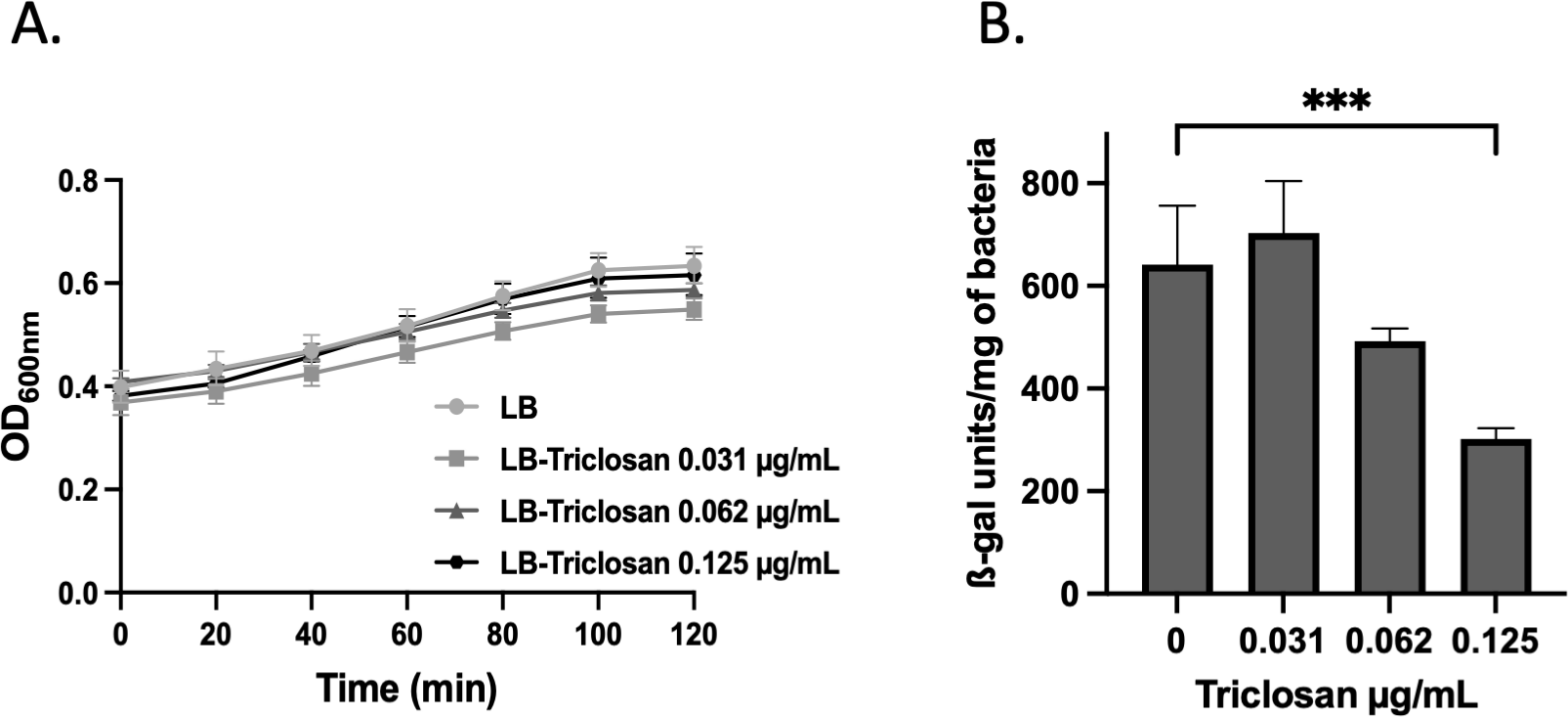
Triclosan affects the activity of the FNR Fe-S regulator. Activity of the *ydfZ* promoter was assessed by measuring the β-gal activity (Miller units) of the P*ydfZ-lacZ* strains. Culture was grown at 37°C under agitation in LB until OD_600 nm_ = 0.4. Four aliquots of this culture were distributed in separate tubes, and triclosan was added at the indicated concentrations. (**A**) Aliquots of 100 µL of these bacterial suspensions were distributed in a 96-well microplate to follow the growth at 37°C under agitation using a Tecan reader. (**B**) After 2 h of incubation at 37°C, β-galactosidase tests were performed. The data presented are the means of three biological replicates, and standard errors of the mean are indicated. Statistical significance was determined using one-way ANOVA with post hoc multiple comparisons performed via the Dunnett test; ***, *P* < 0.005.

Antibiotics targeting the FASII biosynthesis pathway have been shown to modify the acyl-ACP pool. In particular, triclosan, a FabI inhibitor, depletes holo-ACP and long-chain acyl-ACP species (37). We therefore examined FNR maturation using a P*ydfZ-lacZ* reporter strain (Fig. 6B). After growth in LB medium, cells were treated with sub-lethal triclosan concentrations ranging from 0.031 to 0.125 µg/mL (MIC = 0.5 µg/mL). We monitored growth in parallel to rule out fitness-related interference with reporter activity and observed no growth difference between the untreated and the triclosan-treated cultures (Fig. 8A). Triclosan induced a dose-dependent decrease in *PydfZ-lacZ* activity, with a 2-fold reduction observed at 0.125 µg/mL compared to the untreated control (Fig. 8B).

Our results showed that external disruption of ACP homeostasis via FASII inhibition specifically impairs FNR maturation, thereby demonstrating that the integrity of fatty acid biosynthesis is intimately linked to the functionality of the Fe-S cluster assembly machinery.

## DISCUSSION

Our study revealed a connection between two essential and ancient cellular processes, fatty acid metabolism, and [Fe-S] cluster biogenesis. We identified the molecular basis of this connection by uncovering a physical interaction between ACP, the acyl carrier protein that provides an acyl chain to the fatty acid biosynthetic pathway, and IscS, the cysteine desulfurase that provides sulfur for [Fe-S] biogenesis. We showed that ACP levels directly influence the activity of cellular [Fe-S]-bound proteins, whether they are transcriptional regulators, metabolic enzymes, or respiratory chain components.

Using a combination of Boltz modeling, bacterial two-hybrid assays, co-purification experiments, and *in vitro* binding assays, we demonstrated that ACP interacts with the IscS cysteine desulfurase, enhancing its enzymatic activity. ACP is initially produced as an apo-form and undergoes two maturation steps. It is first modified by the covalent attachment of a 4′-phosphopantetheine group to its serine residue at position 36 (holo-ACP) and subsequently acylated with a malonyl moiety. While as-purified ACP, which is expected to comprise equal proportions of apo- and holo-forms and a small fraction of the acylated form (22), binds to IscS *in vitro*, we found that only the acylated form of ACP exerts a positive effect on IscS activity. Both the maturation state of ACP and the specific acyl chain attached to it are expected to reflect the activity of the fatty acid biosynthesis machinery (37). Hence, it is tempting to propose that the IscS/ACP interaction allows the level of available fatty acids to fine-tune [Fe-S] biogenesis efficiency and eventually serves as a strategy to coordinate the cellular levels of fatty acids and [Fe-S] clusters.

Computational modeling, mutagenesis, and biochemical analyses suggest that the interaction between ACP and IscS involves negatively charged residues on ACP (Asp_35_ and Asp_38_) and positively charged ones on IscS (Arg_112_ and Arg_116_). Moreover, the computational model indicates that the ACP attached phosphopantetheine group may fit into a hydrophobic pocket at the interface of two IscS monomers, thereby enhancing the stability of the IscS₂-ACP₂ complex. Consistently, the ACP Ser_36_ residue, which is positioned in the vicinity of Asp_35_ and Asp_38_ residues and carries the 4′-phosphopantetheine group, is crucial for ACP binding to IscS. A prior study showed that DTT disrupts ACP-IscS co-precipitation, implying a disulfide bond may stabilize the complex during purification (14). Our structural model supports this: upon ACP binding, the IscS S-loop rotates, positioning Cys328 within 2.6 Å of the phosphopantetheine thiol, enabling disulfide bond formation. This covalent linkage likely preserves the ACP-IscS complex during purification. Furthermore, the stimulatory effect of ACP on IscS activity requires its acylated form. Despite *in vitro* acylation attempts, we could not obtain pure acylated ACP. Using purified ACP synthase to first convert ACP to its holo-form before acylation following established methods, may improve yields (38). This optimized ACP preparation will be used in future studies.

**Fig 9:**
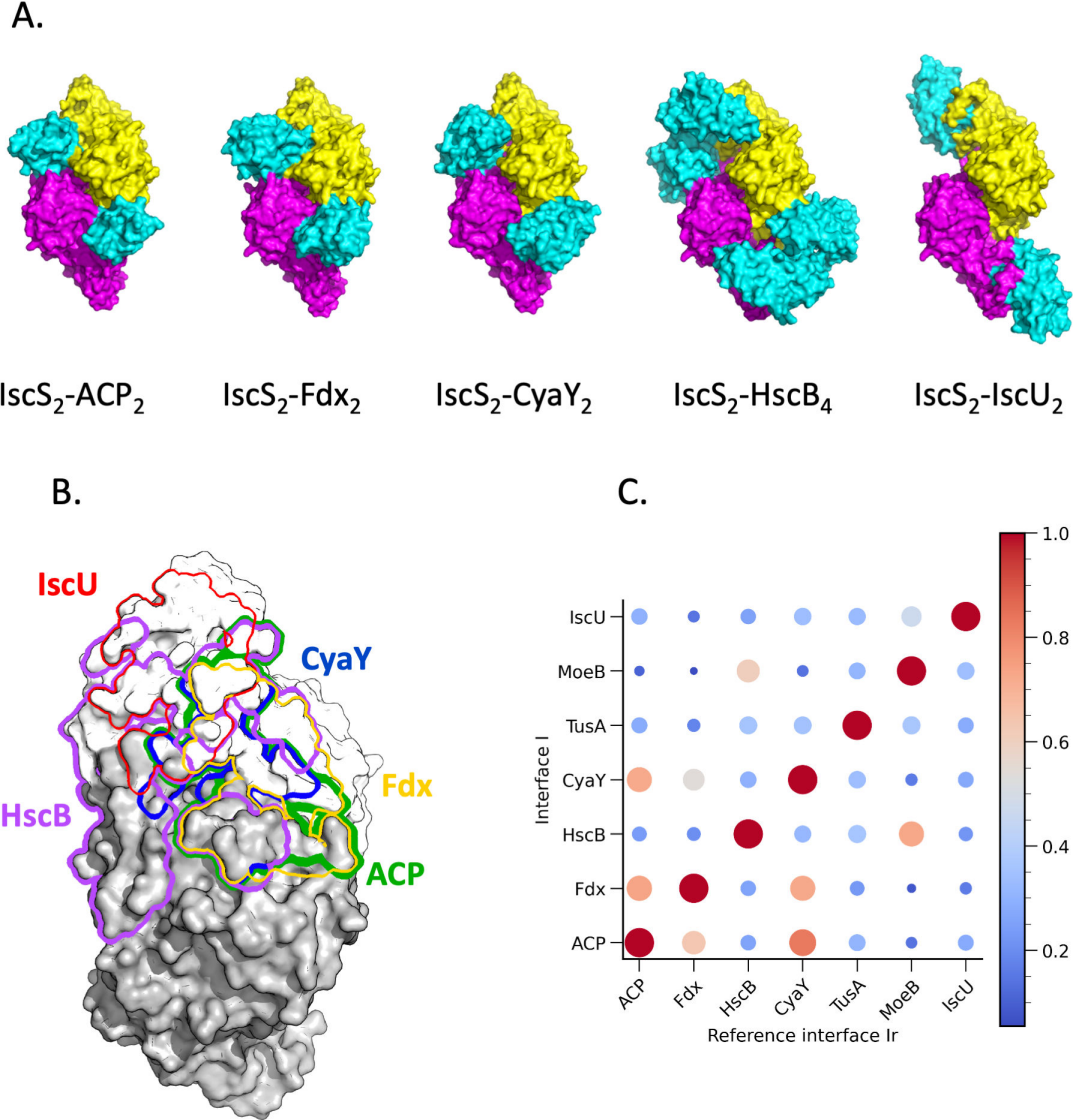
Structural predictions of IscS with its interactants. (**A**) Predictions of the structures of IscS-based complexes were made using Boltz-2. IscS dimers (in yellow and magenta) are shown in interaction with ACP, Fdx, CyaY, HscB, and IscU (in cyan). The stoichiometries of the complexes are given in the figure. (**B**) Footprints of the proteins ACP (green), Fdx (yellow), CyaY (blue), HscB (magenta), and IscU (red) on the dimer of IscS in light and dark gray. (**C**) Quantification of interface overlaps for ACP, Fdx, HscB, CyaY, TusA, MoeB, and IscU with IscS by a Jaccard index on common interface atoms. One means a full overlap, whereas 0 means no overlap. Overlaps between ACP and Fdx/CyaY are 0.7366 and 0.7197, respectively, whereas the overlaps between ACP and the other IscS partners are much lower (less than 0.3).

Interestingly, the triad Ser_36_, Asp_35_, and Asp_38_ is located within the same α2 helix that contains the binding site for fatty acid biosynthesis enzymes FabA, FabI, and FabG (39–41). Conversely, on the IscS side, the Arg_112_ and Arg_116_ residues locate within the region involved in the interaction with several IscS partners. In particular, Arg_116_ is crucial for IscS binding to CyaY an effector of ISC-dependent [Fe-S] biogenesis (42, 43). Moreover, Boltz-2-generated structural predictions of IscS and its partners revealed that Fdx, CyaY, and ACP share overlapping binding regions on IscS, suggesting that ACP targets a hub region shared with these proteins (Fig. 9A through C). Whether this spatial overlap and potential steric hindrance mediate ACP-dependent regulation of Fe-S biosynthesis remains to be determined.

In mitochondria, ACP and NFS1 interact via an intermediate protein, ISD11, yielding a stable ACP-ISD11-NFS1 complex (44). In the yeast model, ACP was also shown to potentiate [Fe-S]-dependent aconitase activity (12). By using the Foldseek search method, we failed to identify structures similar to ISD11 (a LYRM protein) within the AlphaFold database, including bacterial proteins (45). Additionally, a BLAST search using the ISD11 sequence yielded no matches in the *E. coli* sequence database and only one hit in prokaryotic databases: a LYR motif-containing protein in *Kocuria palustris*. We therefore concluded that a LYR motif-containing protein (ISD11) mediating NFS1/ACP interaction is not to be found in *E. coli*. Furthermore, our *in vitro* binding assays indicate that ACP directly binds to bacterial IscS, pointing to a major difference between the bacterial and eukaryotic situations. However, it is noteworthy that two out of four residues of NFS1 involved in interaction with ISD11 are conserved in IscS, while ACP residues involved in interaction with ISD11 in eukaryotes are conserved in *E. coli* ACP (Fig. S9, blue stars) (10). The evolutive advantage, if any, of the presence of such residues in the absence of ISD11 remains unclear. Conversely, although the key residues of *E. coli* ACP required for its interaction with IscS are conserved in eukaryotes, only one of the two arginine residues from IscS involved in ACP binding is conserved in NFS1 (Fig. S9, red stars). This difference may explain why NFS1 is unable to bind ACP directly.

ACP plays a central role in fatty acid biosynthesis, acting as a carrier for fatty acids during their elongation within the FASII cycle. The question arises of what is the teleonomic advantage of coupling fatty acid biosynthesis and [Fe-S] biogenesis. In fact, there are several proteins whose role and/or function depend upon both fatty acid and [Fe-S] biology and which could benefit from a coordinated regulation of [Fe-S] cluster and fatty acid syntheses. A first convergent node is BioB, an enzyme catalyzing the synthesis of biotin, an essential vitamin. BioB is a radical SAM enzyme hosting a [4Fe-4S] and a [2Fe-2S] cluster. It intervenes at the last step of a pathway fed by ACP-bound intermediates (Fig. S7A) (35, 46). Our results showed that decreased ACP levels decreased BioB activity, suggesting that the positive effect of acylated ACP on [Fe-S] biogenesis could help coordinate both the levels of [Fe-S] clusters and fatty acid precursors for optimizing BioB activity. A second convergent node is LipA, an enzyme catalyzing the synthesis of lipoate, an essential cofactor. Like BioB, LipA is a radical SAM enzyme hosting two [4Fe-4S] clusters, acting downstream a series of steps involving ACP-bound intermediates (46). Having LipA activity being optimized by a coordination between the level of [Fe-S] and precursors is a reasonable hypothesis. A third convergent node arises with IspG and IspH, two [4Fe-4S] enzymes, required for the synthesis of isopentenyl phosphate, precursor of lipid II and LPS (47). In this case, coordination between fatty acids and [Fe-S] cluster biogenesis could be a way to synchronize cell elongation, as both feed into membrane and cell wall biogenesis. In this context, it is noteworthy that ACP has been shown to interact with MukB and to be essential for its ATPase activity. MukB is a core component of the structural maintenance of chromosomes complex, which plays a crucial role in chromosome segregation during cell division (18, 48). Hence, our study revealing a coordination between ACP and another central process, such as [Fe-S] biogenesis, points to the possibility of ACP acting as a cellular hub integrating homeostasis-regulating signals to coordinate cell cycle progression, division, and proliferation.

## MATERIALS AND METHODS

### Media and growth conditions

Bacterial strains were grown in LB (Lysogeny Broth) rich medium at 37°C under agitation. The following antibiotics were added when necessary: ampicillin (Amp, 100 µg/mL), kanamycin (Kan, 50 µg/mL), chloramphenicol (Cm, 10 µg/mL), and anhydrotetracycline (AnTet) for inducible expression from the Ptet promoter. For iron chelation, 2,2′-Dipyridyl (DIP, 62 µM) was added to the culture medium. Paraquat was used at 100 µM.

### Bacterial strains and plasmids

*E. coli* K12 MG1655 strain, its derivatives, and plasmids used in this study are listed in Tables 1 and 2, respectively. *E. coli* K12 XL1B strain was used for cloning procedures. Primers, with their sequences and descriptions, are listed in Table 3.

**TABLE 1:** Strains used in this study

Lab ref.	Name	Genotype	Reference
FBE051	MG1655	Wild-type K12 *E. coli*	Barras, lab collection
FBE052	XL1B	*recA*1 *endA*1 *gyrA*96 *thi*-1 *hsdR*17 *supE*44 *relA*1 *lac* [F´ *proAB lacIqZ*ΔM15 Tn10 (Tet^r^)]	Stratagene
FBE054	BTH101	F-, *cya*-99, *araD*139, *galE*15, *galK*16, *rpsL*1 (Str^R^), *hsdR*2, *mcrA*1, *mcrB*1, *relA*1	(20)
FBE001	P*soxS-lacZ*	MG1655 ∆*lacZ* P*soxS::lacZ*	Py
FBE005	P*iscR-lacZ*	MG1655 ∆*lacZ* P*iscR::lacZ*	Py
FBE204	P*ydfZ-lacZ*	MG1655 ∆*lacZ* P*ydfZ::lacZ* (Kan^R^)	Py
FBE227	∆*iscS*	MG1655 ∆*iscS*::kan	This work
FBE228	BL21(DE3)	*E. coli B dcm ompT hsdS*(r_B_^-^m_B_^-^) *gal* (DE3)	(49)
FBE008	∆*iscR* P*iscR-lacZ*	MG1655 ∆*lacZ* ∆*iscR* P*iscR::lacZ*	Py
FBE596	∆*iscUA* P*ydfZ-lacZ*	MG1655 ∆*lacZ* ∆*iscUA* P*ydfZ::lacZ* (Kan^R^)	Barras, lab collection

**TABLE 2:** Plasmids used in this study

Lab ref.	Name	Description	Reference
CRISPR interference			
pEB1976	pdCas9	Cm^R^, p15A ori, Ptet-dCas9	(24)
pEB1977	psgRNA	Amp^R^, ColE1 ori, PJ23119 promoter	(24)
pSF4	psgRNA-ACP	Generated by PCR with ebm1822-ebp8	This study
Protein purification			
pEB1188	pET-6His-TEV	Amp^R^, ColE1 ori, T7 promoter	(50)
pSF19	pET-6His-TEV-*iscS*	Insertion of OSD56-57 in pET-6His-TEV (*Eco*RI/*Xho*I)	This study
pSF51	pET-6His-TEV-*acpP*	Insertion of OSD146-147 in pET-6His-TEV (*Eco*RI/*Xho*I)	This study
pSM61	pET-6His-TEV-*acpP_D35N_*	Directed mutagenesis on pSF51 using OSD266-267	This study
pEB1161	pET-6His-TEV-*acpP_S36T_*	6His-TEV-*acpP*_S36T_ under the control of the T7 promoter	(23)
pEB0817	pET28-*aas*-6His	*aas*-6His under the control of the T7 promoter	(51**)**
BACTH			
pEB354	pT25link	KmR, p15A ori, Plac	(52)
pEB355	pT18link	AmpR, ColE1 ori, Plac	(52)
pEB375	pT25-ACP	Translational fusion between T25 and ACP	(52)
pTFP43	pT25-ACP_D35N_	Translational fusion between T25 and the ACP_D35N_ allele	This study
pTSF18	pT25-ACP_S36T_	Translational fusion between T25 and the ACP_S36T_ allele	This study
pTFP22	pT25-ACP_D38N_	Translational fusion between T25 and the ACP_D38N_ allele	This study
pTFP42	pT25-ACP_E41Q_	Translational fusion between T25 and the ACP_E41Q_ allele	This study
pTSF33	pT18-IscS-ISD11	Translational fusion between T18 and *iscS* with ISD11 under the control of the Plac promoter	This study
pEB362	pT25-TolB	Translational fusion between T25 and TolB	(52)
pEB541	pT18-IscS	Translational fusion between T18 and IscS	This study
pTSD52	pT18-IscS_R112E_	Translational fusion between T18 and the IscS_R112E_ allele	This study
pTSD53	pT18-IscS_R116E_	Translational fusion between T18 and the IscS_R116E_ allele	This study
pEB594	pT18-SufS	Translational fusion between T18 and SufS	This study
pEB356	pT18-PAL	Translational fusion between T18 and PAL	(52)
Protein co-purification			
pEB602	pBAD24-CBPlink	AmpR, pBR322 ori, P*bad*	(52)
pEB540	pBAD24-CBP-ACP	Translational fusion between CBP and ACP	(14)
pEB797	pBAD24-CBP-ACP_S36T_	Translational fusion between CBP and ACP_S36T_	(52)
pSD53	pBAD24-CBP-ACP_D35N_	Translational fusion between CBP and ACP_D35N_	This work
pSD54	pBAD24-CBP-ACP_D38N_	Translational fusion between CBP and ACP_D38N_	This work
pSD55	pBAD24-CBP-ACP_E41Q_	Translational fusion between CBP and ACP_E41Q_	This work

**TABLE 3:** Primers used in this study

Name	Sequence	Description
BACTH constructions		
OSD56	TATGAATTCATGAAATTACCGATTTATCTCGA	Amplification of IscS coding sequence (*Eco*RI/*Xho*I)
OSD57	AAACTCGAGTGATTCCGATACCGATTAATGAT	
OSD351	AGGGAATTCATGATTTTTTCCGTCGACAAAG	Amplification of SufS coding sequence (*Eco*RI/*Xho*I)
OSD352	AAGCTCGAGTGCCTCCCTGTTATCCCAGCA	
OSD49	CCACTGCAGGTCGACTCTAGAATTCATGAAATTACCGATTTATCTCG	Amplification of *iscS* for Gibson assembly
OSD50	TATGCGACGGACTGCCATTAATAAACCTCCTTTTCCGATACCGATTAATGATGA	
OSD51	GGCTCATCATTAATCGGTATCGGAAAAGGAGGTTTATTAATGGCAGTCCGTCGCA	Amplification of *isd11* for Gibson assembly
OSD52	CTTAGTTATATCGATGAATTGCTCGTTCTGCAGTTACGTGCGTGGCA	
*acpP* mutagenesis		
ebm114	GACCTGGGCGCGGATACTCTAGACACCGTTGAGCTG	ACP_S36T_ mutagenesis
ebm115	CAGCTCAACGGTGTCTAGAGTATCCGCGCCCAGGTC	
OSD266	TGAAGACCTGGGCGCGAATTCTCTTGACACCGTTGAG	ACP_D35N_ mutagenesis
OSD267	CTCAACGGTGTCAAGAGAATTCGCGCCCAGGTCTTCA	
OSD268	CCTGGGCGCGGATTCTCTTAACACCGTTGAGCTGGTAATG	ACP_D38N_ mutagenesis
OSD269	CATTACCAGCTCAACGGTGTTAAGAGAATCCGCGCCCAGG	
OSD270	CGGATTCTCTTGACACCGTTCAGCTGGTAATGGCTCTGGAAG	ACP_E41Q_ mutagenesis
OSD271	CTTCCAGAGCCATTACCAGCTGAACGGTGTCAAGAGAATCCG	
*iscS* mutagenesis		
OSD361	GGTACTGGATACCTGCGAACAGCTGGAGCGCGAAG	IscS_R112E_ mutagenesis
OSD362	CTTCGCGCTCCAGCTGTTCGCAGGTATCCAGTACC	
OSD363	CTGCCGTCAGCTGGAGGAAGAAGGTTTTGAAGTCAC	IscS_R116E_ mutagenesis
OSD364	GTGACTTCAAAACCTTCTTCCTCCAGCTGACGGCAG	
Construction of psgRNA derivatives		
ebm1822	ACTAGTATTATACCTAGGACTGAGCTAGC	Amplification of psgRNA-ACP
ebp8	**CGCTTTCGCGA**GTTTTAGAGCTAGAAATAGCAAGTTAAAATAAGGC	
Construction of pET-6His-Tev derivatives		
OSD56	TATGAATTCATGAAATTACCGATTTATCTCGA	*iscS* coding sequence (*Eco*RI/*Xho*I)
OSD57	AAACTCGAGTGATTCCGATACCGATTAATGAT	
OSD146	GGGCGGAATTCATGAGCACTATCGAA	*acpP* sequence (*Eco*RI/*Xho*I)
OSD147	GGAGCTCGAGTTCACTTACGCCTGGTGG	
qRT-PCR		
16sFor	AAGTTAATACCTTTGCTCATTGAC	16s
16sRev	GCTTTACGCCCAGTAATTCC	
OSD10	CTGGGCGTTAAGCAGGAAGAAG	*acpP*
OSD11	CCAGAGCCATTACCAGCTCAAC	
OSD112	TCTACGACCGTATGTTTACTCAT	*hmpA*
OSD113	CTGGAAGCTGGTGTGCTTCTG	

Deletion mutations were introduced in MG1655 by P1 transduction using KEIO collection or lab strains as donors (53, 54). Transduced strains were verified by PCR using primers pair hybridizing upstream and downstream of the deleted genes.

Standard procedures for preparation of DNA, amplification, digestion, and ligation were used as previously described (55). Transformations were done by electroporation. Plasmids constructed for this study have been checked by sequencing (Eurofins).

Plasmid expressing the *acpP* guide for CRISPRi, psgRNA-*acp* was generated by inverse PCR using the primers ebm1822 and ebp8 (Table 3), as previously described (24). Amplified fragments corresponding to whole plasmids (2,580 bp) were then phosphorylated (T4 Polynucleotide Kinase, Biolabs) at their 5′ extremities and circularized by ligation (T4 DNA ligase, Biolabs). *Dpn*I was then used to remove the template DNA before transformation in XL1B.

For protein purification, PCR fragments (generated from OSD56-57, 1,248 bp, and OSD146-147, 263 bp, respectively) corresponding to IscS- and ACP-coding regions were cloned between *Eco*RI and *Xho*I sites of pET6His-TEV, generating pET6His-TEV-*iscS* (pSF19) and pET6His-TEV-*acpP* (pSF51), respectively.

Construction of the plasmids for BACTH assays, DNA inserts encoding the proteins of interest were inserted into pT25link (pEB354) or pT18link (pEB355) using *Eco*RI/*Xho*I sites. The pT18-IscS-ISD11 plasmid was constructed using Gibson assembly using PCR-amplified IscS (primers OSD49-50), ISD11 (primers OSD51-52), and *Eco*RI/*Xho*I linearized pT18link.

Mutated alleles of *acpP* were obtained by PCR (with the PfuUltra II DNA polymerase) using primers containing the mutations: OSD266-267 for ACP_D35N_, ebm114-115 for ACP_S36T_, OSD268-269 for ACP_D38N_, and OSD270-271 for ACP_E41Q_ (see Table 3 for sequences), and the plasmids with the wild-type *acpP* allele as template. After amplification, template DNA was removed using the *Dpn*I restriction enzyme. The same method was used to obtain mutated alleles of *iscS* using primers OSD361-362 for IscS_R112E_ and OSD363-364 for IscS_R116E_.

### Co-purification assay

Co-purifications on calmodulin beads were mainly performed as previously described (52). We worked with soluble extracts of the MG1655 strain carrying pBAD-CBP derivatives (CBP-ACP fusions with either the wild-type allele or the mutated alleles of ACP). Extracts were prepared from 100 mL of cultures induced by arabinose 0.01% for 1 h. Bacterial pellets were sonicated in 4 mL of calmodulin-binding buffer (10 mM Tris-HCl, pH 8.0, 150 mM NaCl, 0.1% NP40, 1 mM Mg-acetate, 1 mM imidazole, and 2 mM CaCl_2_). After centrifugation (30 min, 20,000 × *g*, 4°C), supernatants (3 mL) were applied to calmodulin beads (40 µL) and incubated for 2 h at 4°C. The beads were then washed three times in 1 mL of calmodulin-binding buffer, resuspended in 50 µL of Laemmli loading buffer, and then heated for 5 min at 95°C. After centrifugation to eliminate the beads, extracts were loaded on SDS-PAGE for Coomassie blue coloration and Western-blot detection of IscS using polyclonal antibodies.

### mRNA extraction

Strains were grown in LB-Amp-Cm supplemented with 0.1 ng/mL AnTet at 37°C with aeration until OD_600nm_ = 1. Cells were pelleted by centrifugation (10 mL) and immediately frozen at −20 °C. RNA extractions were then performed as previously described (56). Briefly, bacterial pellets were resuspended in a buffer containing glucose 20%, Tris-HCl 25 mM, pH 7.6, and EDTA 50 mM. Cells were broken in the presence of glass beads (0.1 mm diameter) and acid phenol (pH 4.5) in a cell disruptor. Successive purification steps with Trizol and chloroform were performed before isopropanol precipitation. Purified RNAs were then treated with DNase I by using the TURBO DNA-free reagent (Ambion) in order to eliminate residual contaminating genomic DNA.

### Gene expression analysis by quantitative qRT-PCR

cDNA synthesis was carried out as previously described (57). Oligonucleotides were designed in order to synthesize 100–200 bp amplicons (Table 3). Quantitative real-time PCRs (qRT-PCRs), critical threshold cycles (CT), and *n*-fold changes in transcript levels were performed and determined using the SsoFast EvaGreen Supermix (Bio-Rad) and normalized with respect to 16S rRNA whose levels did not vary under our experimental conditions. We analyzed the results using the Bio-Rad CFX Maestro software. Assays were performed using quadruplicate technical replicates and repeated with three independent biological samples. The results are presented as the means of the technical replicates, and error bars are the standard errors of the means. Biological replicates were treated independently and did not show any significant variations.

### HPLC analysis of tRNA modifications

To extract total tRNAs, *E. coli* cells (1 L) were grown until OD_600nm_ = 1.5, and tRNAs were extracted as previously described (58). Total purified tRNA (100 mg) was digested by nuclease P1 with a ratio of 2 units nuclease/100 µg of tRNA, overnight at 37°C, followed by alkaline phosphatase treatment. Hydrolyzed tRNAs were analyzed by HPLC as previously described (59). Briefly, digested tRNAs were injected onto a C18 reverse phase HPLC column (Kinetex 4.6 × 250, 100 Å, 5 µm, packing, Phenomenex) pre-equilibrated with 100% Solvent A (2.5% methanol and 10 mM NH_4_H_2_PO_4_, pH 5.3). Nucleosides were eluted using Solvent B (20% methanol and 10 mM NH_4_H_2_PO_4_, pH 5.1) and Solvent C (35% acetonitrile and 10 mM NH_4_H_2_PO_4_, pH 4.6) using a gradient. Nucleosides were detected by following the absorbance at 260 nm, and the retention time of each desired modified nucleoside, such as s^2^C, mnm^5^s^2^U, s^4^U, and ms^2^i^6^A, was determined using a characteristic UV spectrum as previously described (59). The levels of each modified nucleoside are estimated based on the area of each modified nucleoside relative to 100 mg of total tRNAs.

### ß-galactosidase assay

Cells were grown in LB medium at 37°C in biological triplicate. ß-gal activity was determined as previously described (54). Average values of ß-gal unit/mg of bacteria are represented, and error bars correspond to the standard deviation of the means.

### Bacterial adenylate cyclase two-hybrid assay

We used BACTH to test protein-protein interactions as previously described (20, 60). After co-electroporation of the BTH101 strain with the two plasmids expressing the hybrid proteins, cells were spread on LB-Amp-Kan plates. Liquid cultures of clones grown in LB-Amp-Kan-IPTG (0.5 mM) were then spotted on LB-Amp-Kan plates with IPTG 0.5 mM and X-Gal 50 µg/mL plates and incubated at 30°C for 2 days and scanned (SCAN 4000, Intersciences).

### Gentamycin killing assay

Strains were grown aerobically in LB at 37°C to an OD_600nm_ of 2. Cultures were then diluted in LB for an equivalent OD_600nm_ = 1. At this point, gentamycin (5 µg/mL) was added to the cells. Aliquots were taken from the culture at indicated time points, diluted in phosphate-buffered saline solution, and colony-forming units (CFUs) were determined. The CFUs at time point 0 (used as the 100%) were around 10^9^ CFU/mL in all experiments.

### 6His-tagged proteins purification

Recombinant 6His-IscS and 6His-ACP proteins and their derivatives were purified. Briefly, MG1655(DE3)/pET-6His-TEV-*acpP* and MG1655(DE3)/pET-6His-TEV-*iscS* strains were grown in LB (1 L) at 37°C until OD_600nm_ = 0.5, and then, 1 mM IPTG was added to induce fusion protein gene expression. For MG1655(DE3)/pET-6His-TEV-*acpP* and derivatives cultures, 10 mM of pantothenate was added to the culture in order to favor the production of the holo form of ACP (50). Cultures were further incubated at 30°C for 4 h. After pelleting, cells were broken by sonication in 40 mL of buffer A (100 mM Hepes, 150 mM NaCl), and the extracts were then centrifuged at 30,000 × *g* for 45 min at 4°C. Supernatants were loaded onto a 5 mL Ni-nitrilotriacetic acid agarose column equilibrated with buffer A. The column was washed with 20 volumes of buffer A, and the proteins were eluted with an imidazole gradient (30–500 mM). Fractions were pooled and dialyzed overnight at 4°C against buffer A to remove imidazole. Purified recombinant protein quality was checked by SDS-PAGE analysis and quantified using a nanodrop and cognate epsilon values.

### *In vitro* acylation of ACP

The tagged acyl-ACP synthetase, 6His-Aas, was purified as previously described using the pET28-Aas-6his plasmid (pEB0817) (51). Acylation reactions were carried out by incubating 1 µg of purified ACP with 20 µg of palmitate and 0.25 µg of Aas in an acylation buffer (100 mM Tris-HCl pH 8, 0.5 mM DTT, 10 mM ATP, 10 mM MgSO_4_) in a total volume of 20 µL. The reactions were incubated at 37°C for 3 h. The resulting ACP species were then analyzed on a 15% SDS-PAGE stained with Coomassie Blue (23).

### *In vitro* IscS-ACP binding assay

Purified 6His-IscS (15 µM) was incubated with purified 6His-ACP (30 µM) or the ACP variant 6His-ACP_D35N_ and 6His-ACP_S36T_ (30 µM) in a 10 µL reaction mix in purification buffer A (100 mM Hepes, 150 mM NaCl). Reactions were incubated at 37°C for 20 min before migration on a non-denaturing 12% PAGE and staining with Coomassie Blue.

### Cysteine desulfurase enzymatic activity assay

IscS cysteine desulfurase activity was determined using the methylene blue assay as previously described (61). Briefly, sulfide produced by IscS desulfurase activity was monitored as follows: cysteine (1 mM) was added to purified proteins, and the mix was incubated for the indicated times at 37°C in the presence of dithiothreitol (DTT) 2 mM. Reactions were quenched by the addition of *N,N*-dimethyl-*p*-phenylenediamine (DMPD) 2.5 mM and FeCl_3_ 3 mM. After 30 min of incubation at 37°C, the reactions were centrifuged for 5 min at 20,000 × *g* in order to eliminate protein aggregates. Methylene blue formation was monitored at 670 nm in a spectrophotometer. Sodium sulfide, Na_2_S, was used as a standard for calibration.

### Aconitase enzymatic activity assay

Aconitase activity was measured by following the transformation of isocitrate to *cis*-aconitate, which can be monitored at 240 nm in a UV spectrophotometer as previously described (62). Briefly, cells were grown in LB with Amp, Kan, and AnTet (0.1 ng/mL) when specified until OD_600nm_ around 2. Spheroplasts were immediately prepared by incubating cells (around 2.10^9^) in Tris buffer 25 mM, pH 7.8, with sucrose 0.5 M on ice for 10 min, and a further lysozyme treatment (0.2 mg/mL). Spheroplasts were kept at −20°C and treated individually to avoid any time lapse before the aconitase test. After sonication, extracts were immediately incubated in the presence of isocitrate as substrate (50 mM Tris pH 7.8, 0.5 mM MnCl_2_, 20 mM isocitrate) at 30°C, and absorbance at 240 nm was followed during 2 min. For each extract, protein concentration was determined by a Bradford assay. Specific activities were calculated using an extinction coefficient of 3.6 mM^−1^ cm^−1^ for *cis*-aconitate.

### Modeling the ACP_2_-IscS_2_ complex structure with 4′-PP and PLP modifications

The structure of the ACP_2_-IscS_2_ complex was modeled using Boltz-2 (19). Boltz-2 uses multiple sequence alignment (MSA) of the protein sequences to model as input, and we used the automatic MSA generation implemented in Colabfold (63). We modeled the complex with the 4′-PP bound to the serine 36 of the ACP chains, and the PLP bound to the lysine 206 of the IscS chains. For the 4′-PP, we used the ”modification” field of the yaml configuration file of Boltz-2 with the CCD code 4HH (https://www.rcsb.org/ligand/4hh) corresponding to the 4′-PP-serine. Since no CCD is available on the Protein Data Bank for the PLP bound to lysine, we added a new residue coding the PLP bound to lysine into the CCD cache of Boltz-2 as described in the following: https://github.com/benf549/boltz-generalized-covalent-modification/tree/main. We named KPLP the new CCD encoding the PLP bound to lysine. The scripts used to add the KPLP CCD to the CCD cache of Boltz-2 are given in supplementary materials, as well as the yaml configuration file of Boltz-2.

### Structural modeling and interface prediction of IscS partners

Structural predictions for the protein complexes were performed using Boltz-2 (19). Input sequences were prepared using a custom pipeline to systematically pair the scaffold dimer (Chains A and B, corresponding to IscS dimer) with various partner proteins in dimeric forms (Chains C and D) for ACP, Fdx, CyaY, and IscU or tetrameric forms (chains C, D, E, and F) for HscB. The resulting models were output in CIF format for downstream structural analysis. Comparative analysis of the binding modes was conducted using PyMOL (Schrödinger, LLC) (Schrödinger, LLC. The PyMOL molecular graphics system, version 1.8. November 2015) via the pymol2 Python libraries. To facilitate spatial comparison, all predicted complexes were structurally aligned to a common reference scaffold (IscS dimer). The alignment was performed using the cmd.align function, specifically targeting the alpha carbons of the scaffold dimer (Chains A and B), ensuring a fixed orientation for all analyzed partners.

To visualize the spatial distribution of the interactome, molecular “footprints” were projected onto the solvent-accessible surface of the scaffold. A custom delineation pipeline (delineate.py) was employed as follows: for Patch Identification—scaffold residues participating in the interface were identified and used to generate a binary surface mask. For image processing: to produce clean visual boundaries, the raw interface patches were processed using a 2D Gaussian filter (σ = 1.0) to smooth edges, followed by a thresholding step to define the contour limits. For visualization, the scaffold surface was rendered in grayscale to provide a neutral background, and binding site boundaries for different partners were then overlaid as color-coded contours (e.g., green for ACP, blue for CyaY, red for IscU, yellow for Fdx, and purple for HscB) to illustrate the mosaic of the binding surface.

Interface overlaps were quantified by calculating the overlap of contact residues between different partners. The interface for each partner was defined as all scaffold atoms within an 8 Å cutoff distance of the partner protein. The similarity between two interfaces, Si and Sj, was determined using the Jaccard Index (J) (19):


$$J\left(Si,\;Sj\right)\;=\frac{|Si\;\cap \;Sj\;|}{|Si\;\cup \;Sj\;|}$$

where S represents the set of unique atom serial numbers identified within the contact zone. These calculations were automated via a custom script (overlaps.py) to generate a cross-comparison matrix of the interactome.

## SUPPLEMENTAL MATERIAL

Supplemental Material mbio.00930-26-s0001.pdf Supplemental figures and text.

ASM does not own the copyrights to Supplemental Material that may be linked to, or accessed through, an article. The authors have granted ASM a non-exclusive, world-wide license to publish the Supplemental Material files. Please contact the corresponding author directly for reuse.
