# Supplementary material for: A connection between two ancient and essential cellular processes, iron-sulfur protein biogenesis and fatty acid synthesis, in Escherichia coli
Source: mBio. 2026 Jun 15;17(7):e00930-26. doi: 10.1128/mbio.00930-26 (PMC13343975; doi:10.1128/mbio.00930-26)
Supplement: Supplemental Material — Supplemental figures and text. [file mbio.00930-26-s0001.pdf]

Figure S1

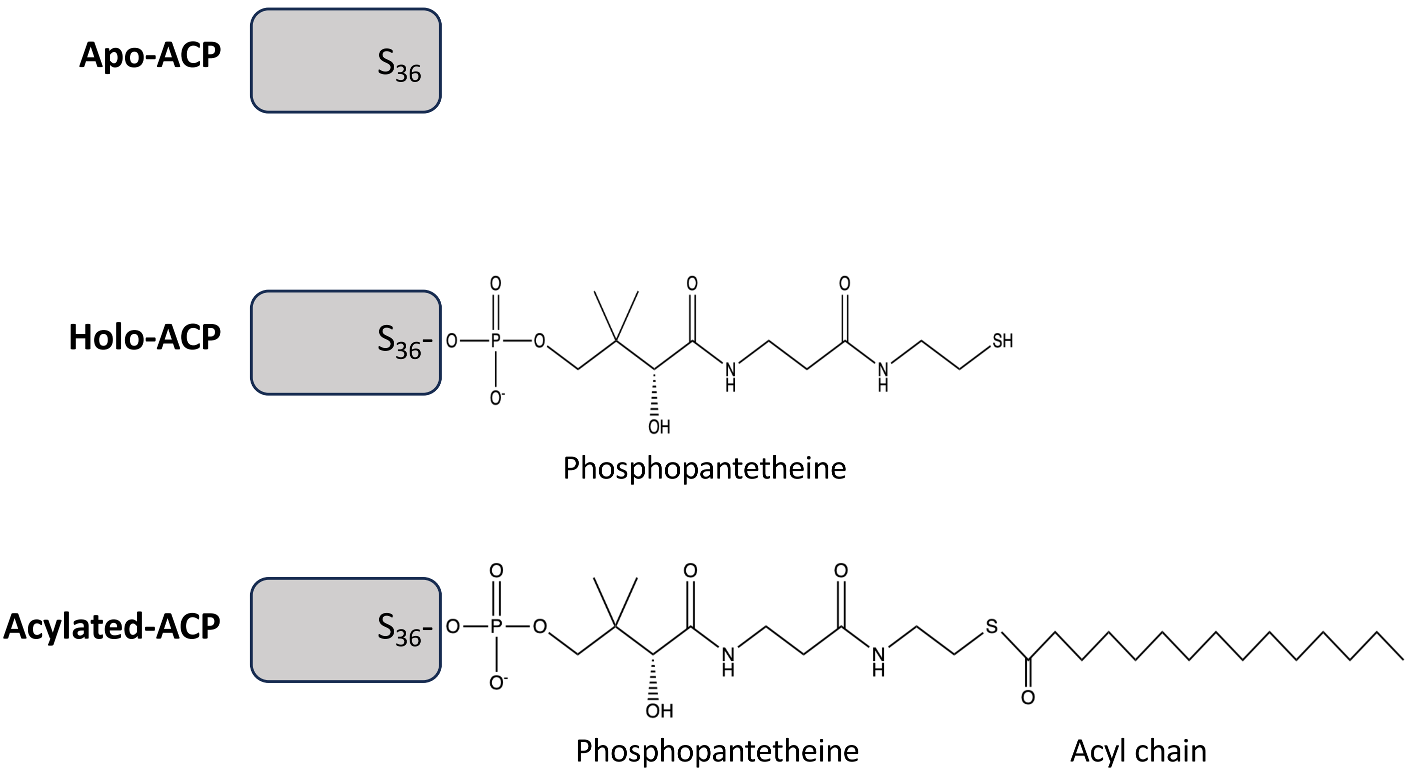

Fig. S1: The three molecular species of ACP found in the cell. ACP is initially synthesized in its apo-form. The first maturation step involves the covalent attachment of a 4'-phosphopantetheine group to the hydroxyl group of serine 36 in ACP via a phosphodiester bond, converting it to holo-ACP. In the second step, a malonyl group is transferred to the terminal thiol group of phosphopantetheine through transesterification. This loaded ACP then transports the malonyl chain to enzymes in the FASII pathway, where it undergoes elongation. The nature of the acyl chain linked to ACP varies depending on the stage of elongation.

Figure S2

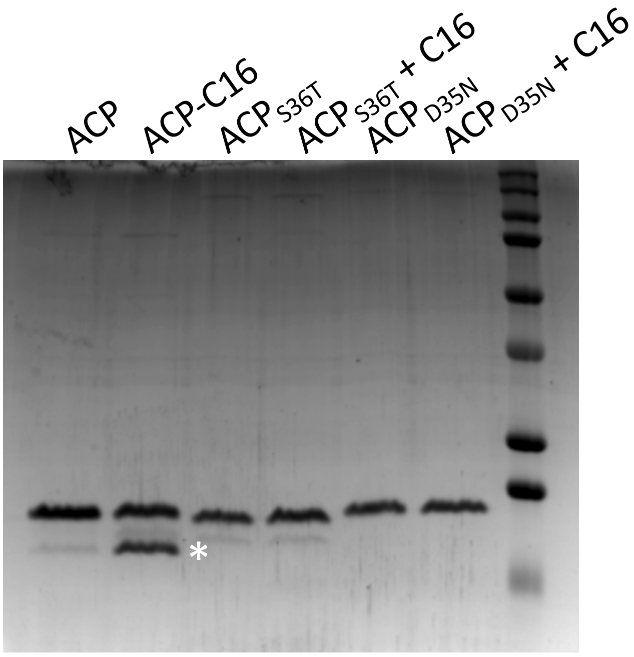

Fig. S2: **ACP variants, ACPS36T and ACPD35N, cannot be acylated in vitro**. Purified 6His-ACP (wild type allele), 6His-ACP$_{S36T}$ and 6His-ACP$_{D35N}$ were treated with palmitate (C16:0) and pure Aas acyl-ACP synthase to catalyze acylation. SDS-PAGE shows the purified ACP and acylated ACP, with the acylated form indicated by an asterisk.

Figure S3

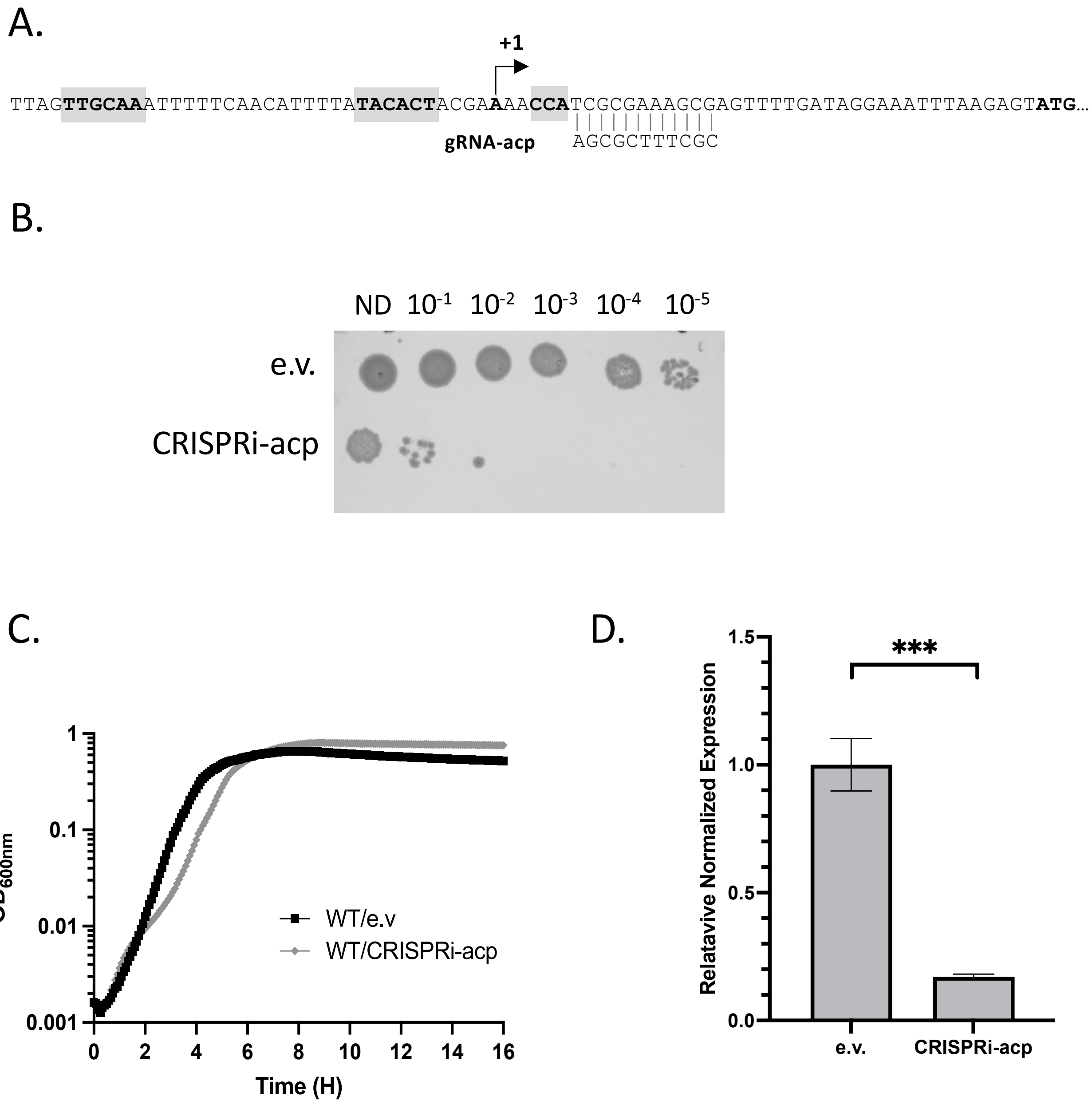

Fig.S3: Validation of the CRISPRi tool to decrease *acpP* expression. **A.** Upstream region of *acpP* coding sequence. Grey boxed sequences upstream the transcriptional start site (+1) indicate -10 and -35 boxes. Grey boxed sequence downstream the +1 indicates the PAM sequence located in front of the complementary sequence of the guide RNA we used (gRNA-*acp*). **B.** MG1655 WT strain was co-transformed with either the pdCAS9 and the psgRNA plasmids (e.v.) or with the pdCAS9 and psgRNA-*acp* plasmids (CRISPRi-acp). Cells were grown in LB until OD_{600nm} = 1 and indicated dilution were spotted (3 µL) on LB plate containing 500 ng/mL AnTet in order to induce dCas9 expression. Plates were incubated one night at 37°C before imaging. **C.** Same strains as described above were cultivated in LB at 37°C in presence of 0.1 ng/mL of AnTet in a microtiter plate reader and OD600nm were recorded. **D.** Same strains as described above were cultivated in LB at 37°C until OD_{600nm} = 1 in presence of 0.1 ng/mL of AnTet. RNA were extracted and treated as described in the Materials and Methods section. Quantitative Real-Time PCR was used to quantify *acpP* cDNA derived from RNA. Expression levels were normalized using 16S rRNA as internal standard and presented as the n-fold change of the CRISPRi-acp strain compared to the e.v. strain. Results are presented as the mean of 4 replicates and standard errors to the means are indicated. ***, P<0.005.

Figure S4

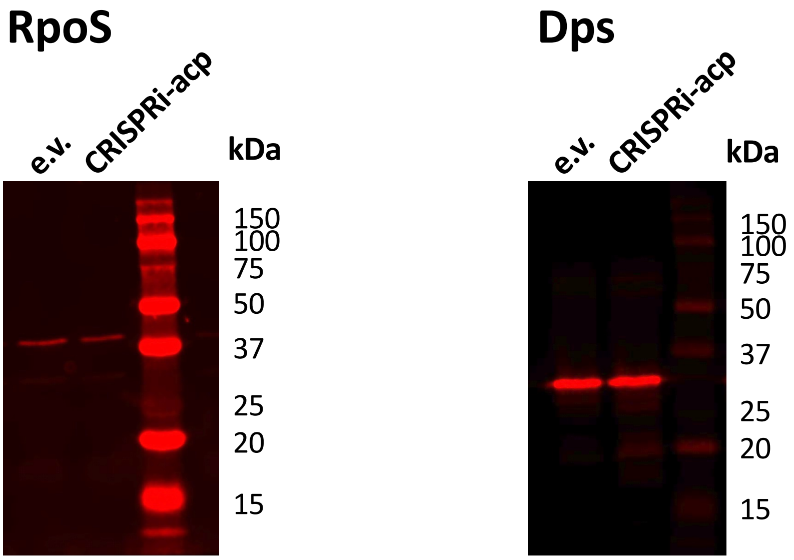

Fig. S4: Moderate decrease of *acpP* expression does not lead to a general stress in the bacterial population. Bacterial strains were grown in LB-Amp-Cm-AnT 0.1 ng/mL until $OD_{600\ nm}$ around 1. Crude extracts were prepared by resuspending cell pellets in1X Laemmli buffer (30 µL/OD_{600nm}=1) and heating 10 min at 100°C. Western blot were performed using polyclonal anti-RpoS antibodies (left panel), or monoclonal anti-flag antibodies to detect Dps-SPA tagged protein (right panel). Fluorescent secondary antibodies were then added and scanning was performed using a Li-Cor Odyssey-Fc imaging system.

Figure S5

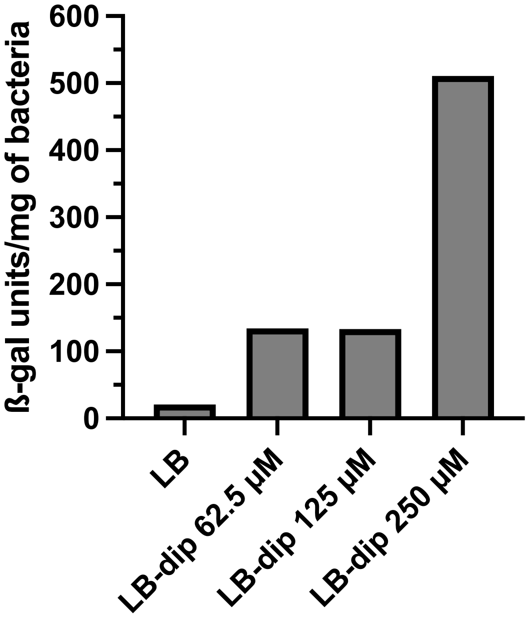

Fig. S5: Study of the *iscR* expression under iron depletion. Bacterial cells, P*iscR-lacZ* strain carrying the CRIPSRi plasmids, pdCas9 and psgRNA (e.v.), were inoculated in LB with increasing concentrations of 2-2'-dipyridyl (DIP) until OD_{600nm} = 2 . Activity of the *iscR* promoter was assessed by measuring the ß-gal activity (Miller units). We noticed that the presence of DIP 62.5 and 125 µM did not alter bacterial growth.

Figure S6

A.

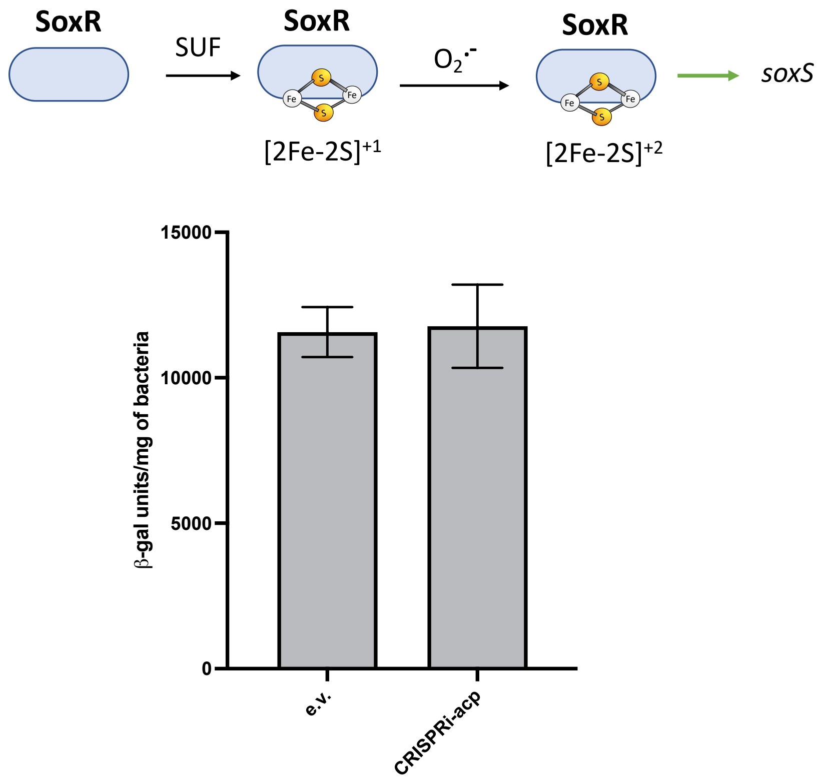

B.

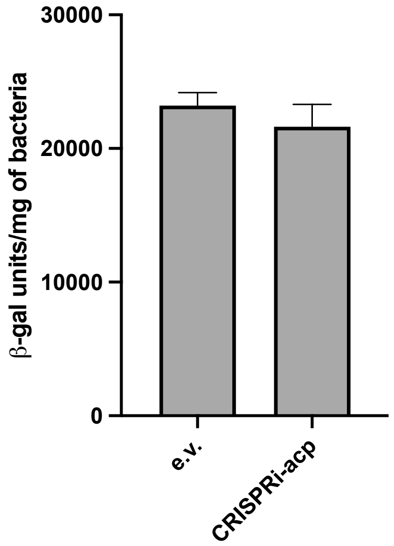

Fig. S6: ACP does not affect the activity of proteins not matured by the ISC system. A. Effect of ACP levels on SoxR-dependent regulation under oxidative stress condition. Activity of the SoxR-*activated soxS* promoter was assessed by measuring the ß-gal activity (Miller units) of the P*sox-lacZ* strain carrying the indicated plasmids in a WT (MG1655) background**.** Strains were grown in LB with 0.1ng/mL of AnTet until OD_{600nm} = 1 and then treated with paraquat dichloride (100 µM) for 1 hour before ß-gal activity was measured. **B. Effect of ACP levels on endogenous β-galactosidase activity**. Wild type MG1655 strain carrying indicated plasmids have been grown in LB with 0.1ng/mL of AnTet and 0.5mM of IPTG until OD_{600nm} = 2.
Represented values are the mean of three independent biological replicates and the error bars represent the standard deviations.

Figure S7

A.

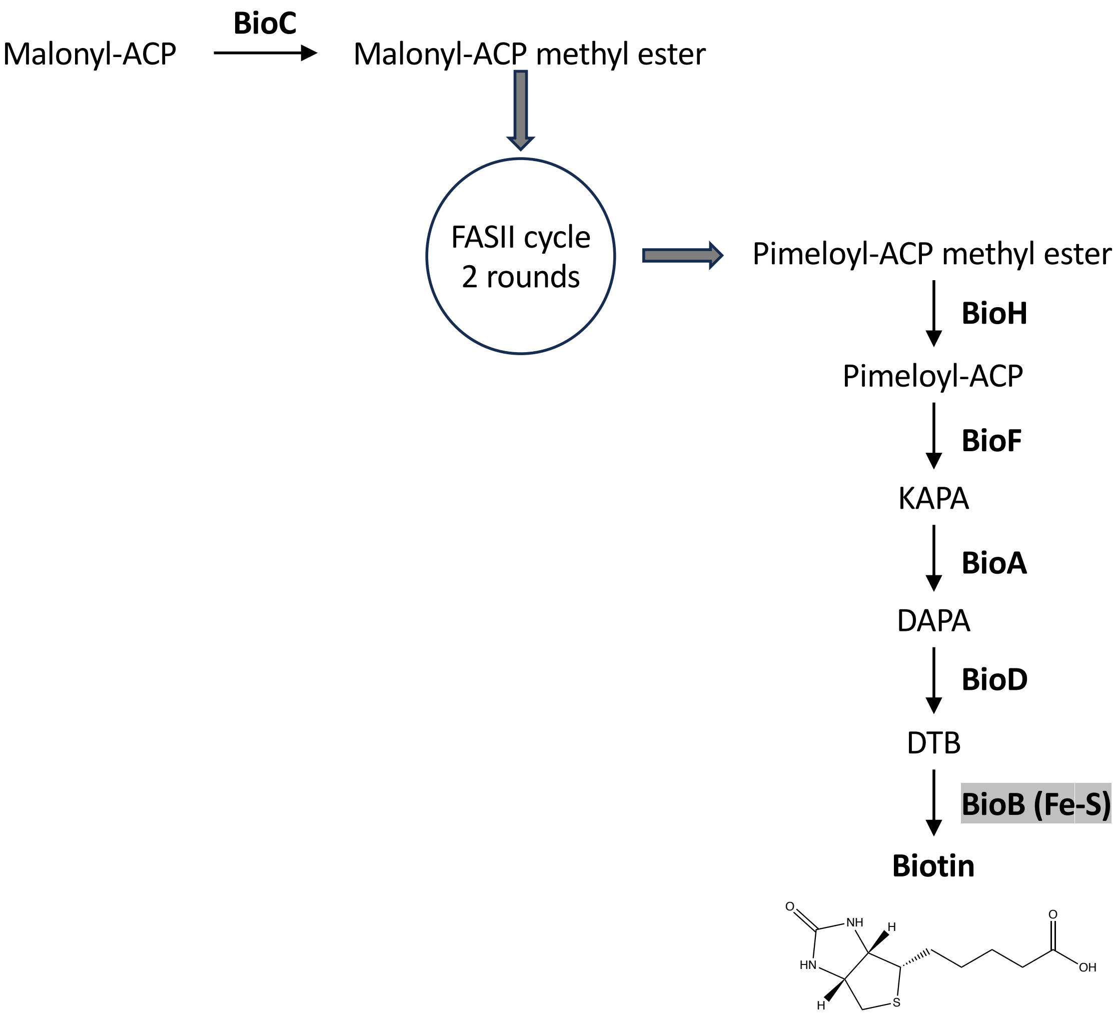

B.

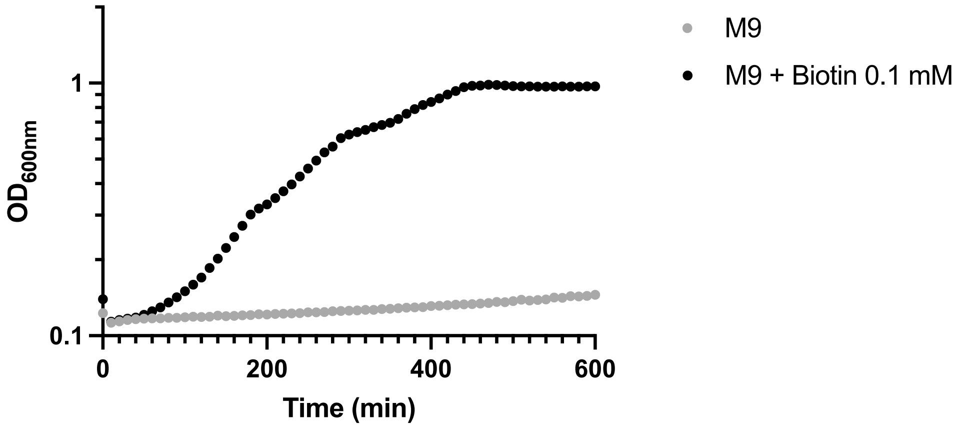

Fig. S7: Biotin is synthesized through a complex pathway from a fatty-acid bound ACP precursor and is essential for bacterial viability. A) *E. coli* biotin biosynthetic pathway. BioB is the radical SAM Fe-S enzyme that catalyses the last step of biotin synthesis by incorporation of a sulfur atom in dethiobiotin (DTB). KAPA: 7-keto-8-amino-pelargonic acid; DAPA: 7,8-diaminopelargonic acid . **B)** Growth of MG1655 Δ*bioD* strain in M9 minimal medium with glucose 0.2 % as carbon source and supplemented (black points) or not (grey points) with 0.1 mM biotin.

Figure S8

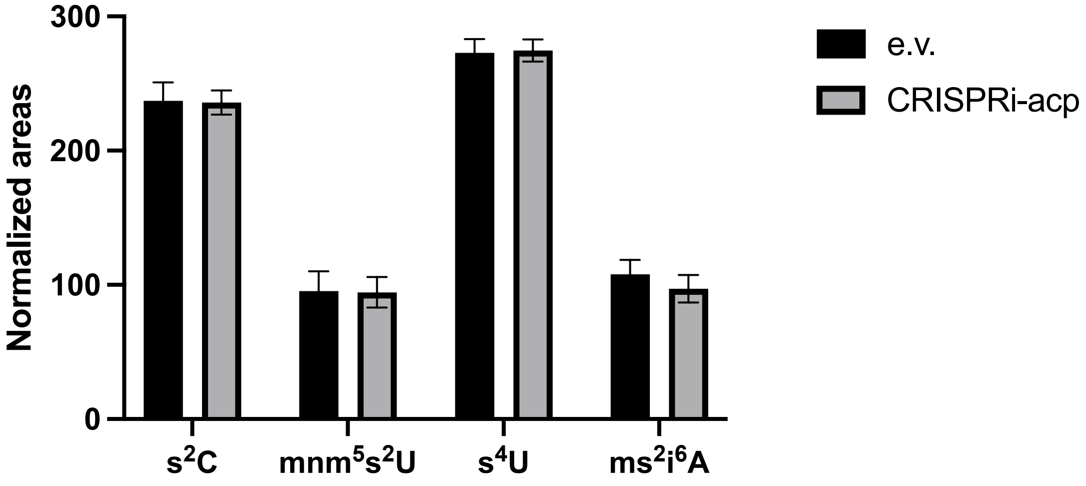

Fig. S8: ACP levels do not impact IscS-dependent tRNA thiolation. Relative levels of specific tRNA modified nucleosides in bacterial cells (MG1655) carrying pdCas9 and psgRNA (e.v., black bars) or psgRNA-*acp* (CRISPRi-acp, grey bars) grown in LB with 0.1ng/mL of AnTet. The retention time of modified nucleosides of s^2C, mnm^5s^2U, s^4U and ms^2i^6A was determined using a characteristic UV spectrum as previously described (1).

The levels of modified nucleosides are estimated based on the area of each modified nucleoside relative to 100 mg of total tRNAs. The data are representative of four independent experiments.

1. C. W. Gehrke, K. C. Kuo, Ribonucleoside analysis by reversed-phase high-performance liquid chromatography. *J Chromatogr* **471**, 3-36 (1989).

Figure S9

A.

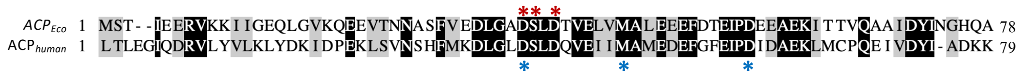

B.

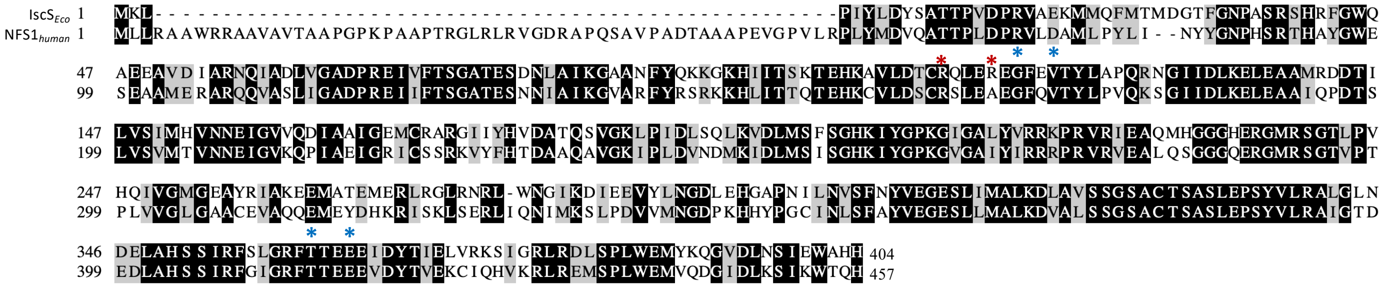

Fig. S9: Comparison of primary sequences of ACP and IscS from *E. coli* and human. A. Amino acid sequence alignment of ACP from *E. coli* (first line) and human ACP (second line) and **B.** IscS from *E. coli* (first line sequence) and Human Nfs1 (second line). Black boxes indicate identical residues, and grey ones, similar residues. Red stars indicate amino acids (for ACP: D_{35}, S_{36}, D_{38} and for IscS: R_{112}, and R_{116}) thought to be involved in ACP-IscS interaction (this work). Blue stars indicate human ACP and Nfs1 residues involved in interaction with ISD11 (D_{35}, M_{44}, D_{56} and R_{72}, D_{75}, E_{314}, Y_{317} respectively) (1).

1. M. T. Boniecki, S. A. Freibert, U. Muhlenhoff, R. Lill, M. Cygler, Structure and functional dynamics of the mitochondrial Fe/S cluster synthesis complex. *Nat Commun* **8**, 1287 (2017).

Supporting information text 1

Boltz-1 scripts

Scripts used to add the KPLP CCD to the CCD cache of boltz-1:

- modified_residues.py: Python scripts to add a new residue into the CCD cache of boltz-1: https://github.com/bougui505/misc/blob/master/singularity/boltz/modified_residues.py. The command line to run the script is:

```
./modified_residues.py -s "CC1=NC=C(COP(O)(O)=O)C(CNCCCCC(N)C(O)=O)=C1O" Ä
-a LYS -o KPLP
```

ACP2holo_PLP_IscS2.yml: yaml configuration file of boltz-1 to build the ACP_2-$IscS_2$ complex structure with 4’PP and PLP modifications:

sequences :
- protein :
id: A
sequence : STIEERVKKIIGEQLGVKQEEVTNNASFVEDL
GADSLDTVELVMALEEEFDTEIPDEEAEKITTVQAAIDYINGHQA
modifications :
- position : 36
ccd: ’4HH ’
- protein :
id: B
sequence : STIEERVKKIIGEQLGVKQEEVTNNASFVEDL
GADSLDTVELVMALEEEFDTEIPDEEAEKITTVQAAIDYINGHQA
modifications :
- position : 36
ccd: ’4HH ’
- protein :
id: [C, D]
sequence : MKLPIYLDYSATTPVDPRVAEKMMQFMTMDGTFGNPASRSHRF
GWQAEEAVDIARNQIADLVGADPREIVFTSGATESDNLAIKGAANFYQKKG
KHIITSKTEHKAVLDTCRQLEREGFEVTYLAPQRNGIIDLKELEAAMRDDT
ILVSIMHVNNEIGVVQDIAAIGEMCRARGIIYHVDATQSVGKLPIDLSQLK
VDLMSFSGHKIYGPKGIGALYVRRKPRVRIEAQMHGGGHERGMRSGTLPVH
QIVGMGEAYRIAKEEMATEMERLRGLRNRLWNGIKDIEEVYLNGDLEHGAP
NILNVSFNYVEGESLIMALKDLAVSSGSACTSASLEPSYVLRALGLNDELA
HSSIRFSLGRFTTEEEIDYTIELVRKSIGRLRDLSPLWEMYKQGVDLNSIE
WAHH
modifications :
- position : 206
ccd: ’KPLP ’
